# A comprehensive model of Earth’s magnetic field determined from 4 years of Swarm satellite observations

**DOI:** 10.1186/s40623-018-0896-3

**Published:** 2018-08-10

**Authors:** Terence J. Sabaka, Lars Tøffner-Clausen, Nils Olsen, Christopher C. Finlay

**Affiliations:** 1 Geodesy and Geophysics Laboratory, NASA Goddard Space Flight Center, Greenbelt, MD USA; 2 Division of Geomagnetism, DTU Space, Technical University of Denmark, Diplomvej, 2800 Kongens Lyngby, Denmark

**Keywords:** Geomagnetism, Field modeling, *Swarm* satellites, Tides

## Abstract

The European Space Agency’s three-satellite constellation *Swarm*, launched in November 2013, has provided unprecedented monitoring of Earth’s magnetic field via a unique set of gradiometric and multi-satellite measurements from low Earth orbit. In order to exploit these measurements, an advanced “comprehensive inversion” (CI) algorithm has been developed to optimally separate the various major magnetic field sources in the near-Earth regime. The CI algorithm is used to determine *Swarm* Level-2 (L2) magnetic field data products that include the core, lithospheric, ionospheric, magnetospheric, and associated induced sources. In addition, it has become apparent that the CI is capable of extracting the magnetic signal associated with the oceanic principal lunar semidiurnal tidal constituent $M_2$ to such an extent that it has been added to the L2 data product line. This paper presents the parent model of the *Swarm* L2 CI products derived with measurements from the first 4 years of the *Swarm* mission and from ground observatories, denoted as “CIY4,” including the new product describing the magnetic signal of the $M_2$ oceanic tide.
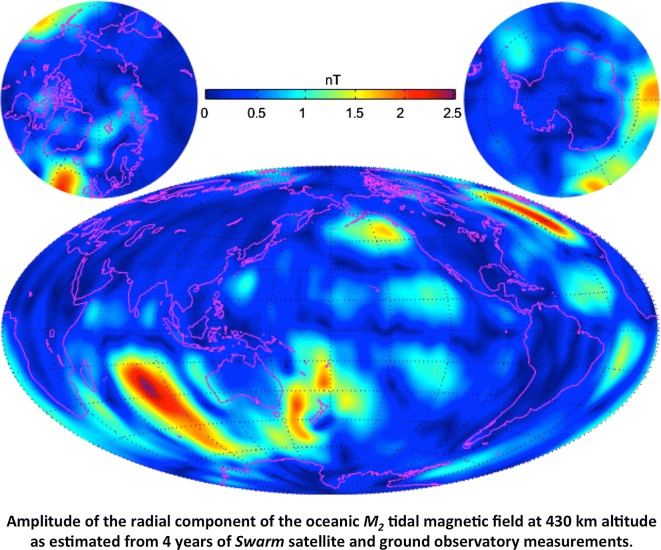

## Introduction

It has been over 4 years since the launch of the European Space Agency (ESA) *Swarm* mission on November, 22, 2013 whose objective is to provide the best-ever survey of Earth’s magnetic field. The constellation of the polar-orbiting trio of satellites was designed to provide north–south gradient information from each spacecraft and unique east–west gradient information from its low-altitude pair of fliers. The orbital planes of the high-altitude flier, known as “*Swarm Bravo*,” and the low pair, known as “*Swarm Alpha*” and “*Swarm Charlie*,” simultaneously sweep out different local times for improved determination of time-varying external fields. In order to best extract the signals from the various magnetic field sources, a modeling approach called “Comprehensive Inversion” (CI) (see Sabaka et al. 2013) has been developed over the years which basically parameterizes all of the major sources and subsequently co-estimates them in order to obtain a proper separation while taking into account systematic errors or biases, which are often more detrimental than random errors. This approach has led to the well-known series of “Comprehensive Models” (CMs) (e.g., Sabaka et al. 2002, 2004, 2015) and has been selected for deriving a consistent set of *Swarm* Level-2 (L2) magnetic data products. The latest CI model, denoted as “CIY4,” is derived from 4 years of *Swarm* magnetic measurements as well as ground-based observations and serves as the source of the fourth version of the L2 data products.

The *Swarm* “Satellite Constellation Application and Research Facility” (SCARF) has been established with the goal of deriving L2 products by combination of data from the three satellites and of the various instruments (Olsen 2013). SCARF uses Level-1b (L1b) data products (which are calibrated time series of magnetic field observations) and auxiliary data in order to determine specific L2 data products. The magnetic data products include models of the core, lithospheric, nonpolar ionospheric and large-scale magnetospheric fields derived using two independent chain branches: several Dedicated Inversion (DI) chains (e.g., Rother et al. 2013; Thébault et al. 2016; Chulliat et al. 2016) in which the various sources are determined in a sequential approach after removing models describing the other sources, and the CI chain where the various data products are co-estimated.

Tyler et al. (2003) were the first to determine the magnetic signal of the oceanic principal lunar semidiurnal constituent $M_2$ from CHAMP satellite measurements, after filtering the data on an orbit-by-orbit basis (which unfortunately removes a significant part of the signal). However, such data pre-processing is not necessary in the CI approach, which successfully extracted $M_2$ from CHAMP data in the CM5 model (Sabaka et al. 2015). Subsequently, CI was used to extract $M_2$ again from the first 20.5 months of *Swarm* data within the context of a model denoted as “CI1” (Sabaka et al. 2016). Encouraged by these results, the SCARF CI software was updated to include $M_2$ extraction that was consequently used to produce the second, third, and fourth year CI *Swarm* L2 data product versions. The original list of L2 products does not include the oceanic $M_2$ field; however, the *Swarm* “Data, Innovation and Science Cluster” (DISC), an international consortium of expert partners with the goal of enhancing the scientific return of the *Swarm* satellite mission by identifying and deriving new, innovative data products, considered the $M_2$ field determined by CI mature enough to be distributed to the broader scientific community. Thus, the CI $M_2$ product is now part of the L2 portfolio and is also described in this paper. It should be noted that unlike the other CI products that have DI redundancy, the $M_2$ product is only produced under the CI chain.

This paper reports on the CIY4 model and the associated L2 magnetic field products, including the new $M_2$ field. Although there have been reports on the DI products and the $M_2$ tidal portion of the CI1 model in the literature (see references above), this is the first complete description of a CI parent model derived from *Swarm* satellite constellation data. This paper first presents a description of the data selection procedure in section “Data selection” followed by a brief overview of the CI algorithm in section “Methodology,” including model parameterization and the estimation procedure, and ends with a discussion of the results in section “Results and discussion,” focusing in particular on the new $M_2$ magnetic field product.

## Data selection

The *Swarm* data used in the CIY4 model is from the *Swarm* Mag-L L1b data product, version 0503, and its selection follows that of previous modeling efforts (e.g., Olsen et al. 2014; Olsen 2015; Finlay et al. 2016). Regarding magnetic activity level, data were chosen only when $Kp \le 3^0$ and $\left| {dDst/dt}\right| \le 3\,\hbox {nT/h}$. Gross outliers were controlled by selecting only those scalar and vector measurements for which the scalar $\Delta F$ and vector $\Delta {\mathbf {B}}$ residuals with respect to the CHAOS-6-x4 model (Finlay et al. 2016) satisfy $\left| {\Delta F}\right| \le 100\,\hbox {nT}$ and $\left| {\Delta \mathbf {B}}\right| \le 500\,\hbox {nT}$. The vector field measurements were further restricted to regions where the sun was more than $10^{\circ }$ below the horizon and whose quasi-dipole (QD) latitude was equatorward of $55^{\circ }$. Interestingly, the vector field measurements have been limited to the quieter conditions of $Kp \le 2^+$ and $\left| {dDst/dt}\right| \le 2\,\hbox {nT/h}$ in other studies (e.g., Sabaka et al. 2016), but the potential negative impact in CIY4 of the additional data from the relaxed selection criteria has been found to be negligible and in fact they may be beneficial since data coverage is improved. Temporal selection of *Swarm* data was between December 1, 2013, to December 31, 2017, at a $15\,\hbox {s}$ sampling rate with north–south (NS) sums and differences being taken between every other pair. The east–west (EW) sums and differences are produced between *Alpha* and *Charlie* from April 17, 2014, to December 31, 2017, when the satellite pair were in a proper configuration. The EW measurements are constructed when *Alpha* and *Charlie* are at equal geographic latitude at slightly different times, usually within $10\,\hbox {s}$.

It should be mentioned that to the authors’ knowledge, only the CI algorithm incorporates data measurement sums (the complement of the differences) (see Sabaka et al. 2013, 2015, 2016) as opposed to field measurements alone (see Olsen et al. 2014), difference measurements alone (see Olsen et al. 2017), and field and difference measurements (see Olsen 2015; Finlay et al. 2016). The sums balance the influence of the differences in determining fields from sources such as the ionosphere.

To complement the *Swarm* measurements, and to provide surface data control, vector hourly mean measurements from permanent magnetic observatories have been included in CIY4. These “observatory hourly means” (OHMs) were selected under the activity conditions of $Kp \le 2^+$ and $\left| {dDst/dt}\right| \le 2\,\hbox {nT/h}$ at all geomagnetic latitudes from December 1, 2013, to October 16, 2017. Note that these criteria are currently more restrictive than those used for the satellites. However, the more relaxed criteria will be investigated for all data in future CI models. Further details on this OHM data set can be found in Macmillan and Olsen (2013).

**Fig. 1:**
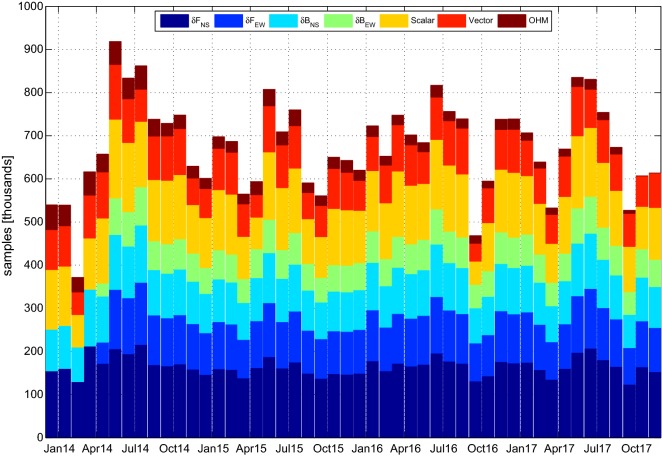
Data distribution for CIY4 over its time domain showing the *Swarm* north–south $(\delta F_{NS})$ and east–west $(\delta F_{EW})$ scalar difference/sum pairs, the north–south $(\delta \mathbf {B}_{NS})$ and east–west $(\delta \mathbf {B}_{EW})$ vector difference/sum pairs, the single scalar and vector *Swarm* measurements, and the vector OHM measurements

A plot of the *Swarm* and OHM data distributions over time used in CIY4 is shown in Fig. 1. Specifically, the plot shows the *Swarm* NS $(\delta F_{NS})$ and EW $(\delta F_{EW})$ scalar difference/sum pairs, the NS $(\delta \mathbf {B}_{NS})$ and EW $(\delta \mathbf {B}_{EW})$ vector difference/sum pairs, the single scalar and vector *Swarm* measurements, and the vector OHM measurements. The side-by-side constellation of the lower pair, *Alpha* and *Charlie*, has been maintained since April 17, 2014, and hence EW differences/sums are used only from this date onwards. Otherwise the data amounts and ratios are fairly consistent with natural variations due to the selection criteria, i.e., due to variations in the *Kp* and *Dst* indices as well as the drifts of the satellite orbital planes through local time; *Alpha* and *Charlie* cover all local times in about 19 weeks, whereas *Bravo* covers all local times in 20 weeks. The OHMs are also absent during the last two months of the data envelope.

## Methodology

### Model parameterization

The CI algorithm considers several major field sources including the core, lithosphere, oceanic $M_2$ tidal, ionospheric and magnetospheric and their associated induced fields, and observatory biases, which account for local baseline field levels, particularly in the local lithosphere. The parameterizations of the various sources are summarized in Table 1.

#### Core and lithospheric fields

The spherical harmonic (SH) truncation level of the internal potential field is $N_{\max }=100$, where the first 16 degrees have secular variation (SV) in the form of order-4 B-splines spanning 2013.9 to 2018.0 with knots every 6 months giving a total of 12 parameters per SH coefficient, and for degrees above 16 the coefficients are constant. The expression for the core/lithospheric potential at time *t* and position $\mathbf {r}$, corresponding to Earth-Centered Earth-Fixed (ECEF) spherical coordinates of radius, colatitude, and longitude $(r,\theta ,\phi )$, is given by

1 $$\begin{aligned} V_{cl}(t,\mathbf {r}) &= \mathfrak {R}\left\{ {a \sum _{n=1}^{16}\sum _{m=0}^n\sum _{q=0}^{11}\left( {a\over r}\right) ^{n+1} \gamma _{nq}^m Y_{nq}^m(t,\theta ,\phi )}\right. \\ &\quad \left.+ {a \sum _{n=17}^{100}\sum _{m=0}^n\left( {a\over r}\right) ^{n+1} \gamma _n^m Y_n^m(\theta ,\phi )}\right\} , \end{aligned}$$

where the $\mathfrak {R}\left\{ {\cdot }\right\}$ operator takes the real part of the expression only and $Y_n^m$ is the surface SH of degree *n* and order *m* given by

2 $$\begin{aligned} Y_n^m(\theta ,\phi )=P_n^m(\cos {\theta })\exp {im\phi }, \end{aligned}$$

where *a* is the Earth mean-radius ($6371.2\,\hbox {km}$), $P_n^m$ and $\gamma _n^m$ are the Schmidt semi-normalized associated Legendre function and static complex Gauss coefficient of degree *n* and order *m*, respectively. The time-variable core field is a linear combination of basis functions $Y_{nq}^m(t,\theta ,\phi )$ with associated multipliers $\gamma _{nq}^m$ such that

3 $$\begin{aligned} Y_{nq}^m(t,\theta ,\phi )= \left\{ { \begin{array}{lll} Y_n^m(\theta ,\phi ) &\quad{} {\mathrm{for}} &{} q=0, \\ Y_n^m(\theta ,\phi ) \int _{2015}^t b_q(\tau )\;\hbox {d}\tau &\quad{} {\mathrm{for}} & q>0, \end{array}}\right. \end{aligned}$$

where $b_q$ is the *q*th cubic B-spline of the expansion and the epoch of the expansion is 2015.0. For $n=1{-}16$ this is equivalent to the usual solid harmonic functions with time-dependent Gauss coefficient multipliers of the form

4 $$\begin{aligned} \gamma _n^m(t)=\gamma _n^m(2015)+\int _{2015}^t \dot{\gamma }_n^m(\tau )\;\hbox {d}\tau , \end{aligned}$$

where

5 $$\begin{aligned} \dot{\gamma }_n^m(t)=\sum _{q=1}^{11} \gamma _{nq}^m b_q(t), \end{aligned}$$

and $\gamma _{n0}^m=\gamma _n^m(2015)$. The general complex Gauss coefficient $\gamma _n^m$ is related to the familiar real Gauss coefficients $g_n^m$ and $h_n^m$ by $\gamma _n^m=g_n^m-i h_n^m$, that is, $g_n^m$ is the real part and $h_n^m$ is the negative of the imaginary part.

#### Oceanic $M_2$ field

The oceanic principal lunar semidiurnal constituent $M_2$ has been included in CIY4 with the same parameterization defined in Sabaka et al. (2015, 2016), where the internal potential has a truncation level of $N_{\max }=36$ and each coefficient is sinusoidal in time with a 12.42060122 hour periodicity with time $\Delta t$ rendered with respect to Greenwich phase. The potential at time $\Delta t$ and position $\mathbf {r}$ in the ECEF system is then

6 $$\begin{aligned} V_{M_2}(\Delta t,\mathbf {r})=\mathfrak {R}\left\{ {a \sum _{n=1}^{36}\sum _{m=-n}^n\left( {a\over r}\right) ^{n+1} \tau _n^m Y_{n \omega }^m(\Delta t,\theta ,\phi )}\right\} , \end{aligned}$$

where $\tau _n^m$ is the complex coefficient and

7 $$\begin{aligned} Y_{n \omega }^m(\Delta t,\theta ,\phi )=P_n^m(\cos {\theta })\exp {i(m\phi +\omega _{M_2}\Delta t)}, \end{aligned}$$

with $$\omega _{M_2}=2\pi /12.42060122\,\hbox {rads/h}$$.

#### Ionospheric field

The CIY4 ionospheric and induced parameterization uses quasi-dipole (QD) symmetric basis function (Emmert et al. 2010; Richmond 1995) as in Sabaka et al. (2004, 2015) in order to conform to the conductivity structures found in the *E*-region ionosphere. As in Sabaka et al. (2015) the induced field now reflects a 3-dimensional (3D) conductivity model where a surface layer containing continents and oceans is underline by a 1-dimensional (1D) mantle known as “1D + oceans” (Kuvshinov 2008). The conductance of sea water has been taken from Manoj et al. (2006) and accounts for ocean bathymetry, ocean salinity, temperature and pressure. Conductance of the sediments is based on the global sediment thicknesses given by the map of Laske and Masters (1997) and calculated by a heuristic procedure similar to that described in Everett et al. (2003). The 1D mantle conductivity has been updated with satellite data by Kuvshinov and Olsen (2006).

As in Sabaka et al. (2015), the conductivity structure induces a secondary field in the spectral domain through transfer functions $\mathbf {Q}(\omega )$ at frequency $\omega$. If $\varvec{\epsilon }(\omega )$ and $\varvec{\iota }(\omega )$ are the vectors of complex SH coefficients for the inducing and induced fields, respectively, at frequency $\omega$, then $\varvec{\iota }(\omega )=\mathbf {Q}(\omega ) \varvec{\epsilon }(\omega )$. These complex matrices are dense owing to the fact that they reflect 3D conductivity, which means that a relatively smooth inducing field can create complicated induced field structure. Contrast this with a 1D conductivity where $\varvec{\epsilon }_n^m$ can only induce $\varvec{\iota }_n^m$, thus leading to a diagonal complex $\mathbf {Q}(\omega )$ whose elements are functions of SH degree *n* only. The frequencies chosen correspond to the daily and sub-daily periods of 24, 12, 8, and 6 hours. In addition, these periods are modulated further by an annual and semiannual periodicity and by scaling from the 3-month running average of the $F_{10.7}$ solar radiation index such that these $\mathbf {Q}$ also reflect an infinite conductor at depth to approximate long-period variations.

#### Magnetospheric field

The CIY4 parameterization of the magnetosphere and associated induced fields also follows Sabaka et al. (2015) by discretizing time into bins within which the fields are treated as static external and internal SH expansions in dipole coordinates, respectively. These SH expansions are to degree $N_{\max }=1$ and order $M_{\max }=1$ for internal and external fields in 1 h bins for the selected quiet periods. This results in 27, 542 hourly bins covering 77% of the hours of the model time span from December 1, 2013, to December 31, 2017.

#### Alignment parameters

Finally, the alignment between the vector magnetometer frame (VMF) and the spacecraft common reference frame (CRF) is parameterized in terms of three Euler angles representing rotations around the *x*-axis of the CRF followed about the new *y*-axis and then the new *z*-axis. The angles are treated as static in 10 day intervals.

**Table 1:** CIY4 parameterization

Field source/effect	# Parms	Description		
Core/lithosphere	13,368	*Spatial: * geographic spherical harmonic (SH) $N_{\max }=100$		
		*Temporal: * order 4 B-splines SV, 6 month knot spacing from 2013.9 to 2018.0, epoch 2015.0, up to $N_{\max }=16$		
$M_2$ tidal	2736	*Spatial: * geographic SH $N_{\max }=36$		
		*Temporal:* period of 12.42060122 hours, Greenwich fixed phase		
Ionosphere/induced	5520	*Spatial:* quasi-dipole (QD) frame, underlying dipole SH $N_{\max }=60$, $M_{\max }=12$		
		*Temporal:* annual, semiannual, 24, 12, 8, and 6 h periodicities with $F_{10.7}$ scaling plus induction via a priori 3D conductivity model (“1D + oceans”) and infinite conductor at depth		
Magnetosphere/induced	165,252	Magnetosphere *Spatial:* dipole SH $N_{\max }=1$ *Temporal:* discretized in 1 h bins		
		Induced *Spatial:* dipole SH $N_{\max }=1$ *Temporal:* discretized in 1 h bins		
OHM biases	465	One vector bias for each station in local spherical system		
VFM-CRF alignment	1350	Three *XYZ*-type Euler angles every 10 days for each satellite		
Total	188,691	−		

### Estimation procedure

The parameters discussed in the previous section are estimated via a nonlinear least squares (LS) problem that is solved using an iterative Gauss–Newton (GN) method (Seber and Wild 2003) with linear equality constraints, denoted LSLE-GN, as introduced in Sabaka and Olsen (2006); Sabaka et al. (2013) and applied in Sabaka et al. (2015, 2016). The *k*th step of the algorithm is given by

8 $$\begin{aligned} {\mathrm{LSLE-GN}} \left\{ { \begin{array}{ll} \min _{\varvec{\Delta x}_k} &{} \left| {\mathbf {L}_k^{+}\left( {\varvec{\Delta d}_k-\mathbf {A}_k\varvec{\Delta x}_k}\right) }\right| _2^2+ \\ &{} \sum _{j=1}^{N_q}\lambda _j\left| {\mathbf {F}_j^{-1}\left( {\mathbf {x}_j^{\prime }-\mathbf {x}_k-\varvec{\Delta x}_k}\right) }\right| _2^2 \\ {\mathrm{subject}}\,{\mathrm{to:}} &{} \mathbf {G}\varvec{\Delta x}_k=-\mathbf {G}\mathbf {x}_k \\ &{} \mathbf {x}_{k+1}=\mathbf {x}_k+\varvec{\Delta x}_k \end{array}}\right. , \end{aligned}$$

where $|\cdot |_2$ is the $\ell _2$ norm, $\varvec{\Delta d}_k\equiv \varvec{\Delta d}(\mathbf {x}_k)=\mathbf {d}-\mathbf {a}(\mathbf {x}_k)$ is the residual vector of the data $\mathbf {d}$ with respect to the nonlinear model vector $\mathbf {a}(\mathbf {x}_k)$ evaluated at $\mathbf {x}_k$, $\mathbf {A}_k\equiv \mathbf {A}(\mathbf {x}_k)$ is the Jacobian of the model vector evaluated at $\mathbf {x}_k$, $\varvec{\Delta x}_k$ are the adjustments to the current parameter vector $\mathbf {x}_k$, and $\mathbf {L}_k\equiv \mathbf {L}(\mathbf {x}_k)$ is the square-root factor of the data noise error-covariance matrix ${\mathbf {C}}_k={\mathbf {L}}_k {\mathbf {L}}_k^{\mathrm{T}}$. There are $N_q$ quadratic constraints, where $\mathbf {F}_j$ is the square-root factor of the *j*th *a priori* covariance matrix ${\mathbf {P}}_j^{-1}={\mathbf {F}}_j {\mathbf {F}}_j^{\mathrm{T}}$ that, along with the Lagrange multiplier $\lambda _j$, specifies the deviation of the solution from the preferred *a priori* model vector $\mathbf {x}_j^{\prime }$. The matrix $\mathbf {L}_k^{+}$ is the pseudo-inverse of $\mathbf {L}_k$ which accounts for infinite variances in $\mathbf {C}_k^{+}=\mathbf {L}_k^{+ \mathrm T} \mathbf {L}_k^{+}$. As will be seen, the system is subject to the linear equality constraints

9 $$\begin{aligned} \mathbf {G}\mathbf {x}=\mathbf {0}. \end{aligned}$$

The solution to the *k*th step of LSLE-GN, denoted $\varvec{\widetilde{\Delta x}}_k$, is given by

10 $$\begin{aligned} \varvec{\widetilde{\Delta x}}_k=\varvec{\overline{\Delta x}}_k-{\mathbf {E}}_k^{-1}{\mathbf {G}}^{\mathrm{T}}\left( {{\mathbf {G}}{\mathbf {E}}_k^{-1}{\mathbf {G}}^{\mathrm{T}}}\right) ^{-1}{\mathbf {G}}\left( {{\mathbf {x}}_k+\varvec{\overline{\Delta x}}_k}\right) , \end{aligned}$$

where ${\mathbf {E}}_k={\mathbf {A}}_k^{\mathrm{T}}{\mathbf {C}}_k^{+}{\mathbf {A}}_k+\sum _{j=1}^{N_q}\lambda _j{\mathbf {P}}_j$, and $\varvec{\overline{\Delta x}}_k$ is the unconstrained solution

11 $$\begin{aligned} \varvec{\overline{\Delta x}}_k={\mathbf {E}}_k^{-1}\left[ {{\mathbf {A}}_k^{\mathrm{T}}{\mathbf {C}}_k^{+}\varvec{\Delta d}_k+\sum _{j=1}^{N_q}\lambda _j{\mathbf {P}}_j\left( {{\mathbf {x}}_j^{\prime }-{\mathbf {x}}_k}\right) }\right] . \end{aligned}$$

For the CIY4 model, four LSLE-GN iterations were performed. The starting model was taken from the CI model determined from 3 years of *Swarm* data. Table 2 shows the $\ell _2$ norm of the adjustment vector $\varvec{\Delta x}_k$ in Eq. 8 for each iteration *k* computed with and without the magnetospheric/induced parameters. The size of the adjustments is three orders of magnitude smaller for $k=3$ compared to $k=0$ when the magnetospheric/induced parameters are excluded and one order of magnitude smaller when all parameters are considered. This, along with inspection of the fields at each iteration, leads to the conclusion that the CIY4 estimate has reasonably converged.

**Table 2:** LSLE-GN convergence for CIY4, where $\left| {\varvec{\Delta x}_k}\right|$ is the $\ell _2$ norm of the adjustment vector $\varvec{\Delta x}_k$ in Eq. 8 and “M/I” denotes magnetospheric/induced parameters

Iteration *k*	$\left| {\varvec{\Delta x}_k}\right| _2$ excluding M/I	$\left| {\varvec{\Delta x}_k}\right| _2$
0	4348.027	8351.771
1	135.642	3230.750
2	6.669	1556.125
3	1.997	642.987

#### Error-covariance

The data noise error-covariance matrix $\mathbf {C}_k$ is designed to account for random, zero-mean error in the measurements and theory, but is also augmented, as will be discussed, to allow for bias mitigation which results from systematic error in the theory. In CIY4, the OHM vector components are in the (*North*, *East*, *Center*) or $\textit{NEC}$ local spherical coordinate system and are given isotropic, i.e., the same for each component, uncertainties, $\sigma$, of 7, 4, and $15\,\hbox {nT}$ for observatories with QD latitudes equatorward of $\pm \, 10^{\circ }$, poleward of $\pm \, 10^{\circ }$ and equatorward of $\pm\, 55^{\circ }$, and poleward of $\pm \, 55^{\circ }$, respectively. Single satellite vector measurements are used in the $\textit{BP3}$ orthogonal coordinate system where “*B*” is along the predicted magnetic field direction, “*P*” is in the $\hat{\mathbf{n}}\times {\mathbf {B}}$ direction where $\hat{\mathbf{n}}$ is the unit vector along the CRF *z*-axis, and “3” completes the system. The uncertainties are assumed isotropic at $2.2\,\hbox {nT}$ and attitude error is assumed negligible. The satellite scalar measurements, *F*, are given the same uncertainty. As for the satellite vector sums and differences, they are computed in the $\textit{NEC}$ system and are assigned isotropic uncertainties of $2.2\,\hbox {nT}$ and $0.3\,\hbox {nT}$, respectively. Therefore, the random error contribution to $\mathbf {C}_k$ is diagonal.

The reason $\mathbf {C}_k$ is iteration dependent is because robust estimation in the form “iterative reweighted least squares” (IRLS) with Huber weights (Constable 1988) is used. Here, the *i*th scalar measurement is assigned a Huber weight at the *k*th GN iteration according to

12 $$\begin{aligned} w_{i,k}={1\over \sigma _i^2} \min {\left( {{c\sigma _i\over \left| {e_{i,k}}\right| },1}\right) }, \end{aligned}$$

where $\sigma _i$ is the assigned uncertainty of the *i*th measurement, $e_{i,k}$ is the current residual, and $c=1.5$. Thus, the Huber distribution has a Gaussian core and Laplacian tails. These weights contribute to the diagonal elements of $\mathbf {C}_k^{+}$.

The CI algorithm reduces many types of data, some of which contain large systematic biases in certain parameter subspaces. The biases considered here are driven by the three factors: (1) measurement type, such as scalar or vector, field or difference or sum, (2) sun position being more than $10^{\circ }$ below the horizon (“dark”) or not (“light”), and (3) QD latitude range, equatorward of $\pm\, 10^{\circ }$ (“low”), poleward of $\pm\, 10^{\circ }$ and equatorward of $\pm\, 55^{\circ }$ (“mid”), and poleward of $\pm\, 55^{\circ }$ (“high”). Therefore, in order to mitigate these effects Sabaka et al. (2013) introduced a scheme known as “Selective Infinite Variance Weighting” (SIVW), which introduces additional “nuisance” versions to the usual “nominal” parameters in $\mathbf {x}$ that are intended to absorb this bias. Mathematically, this is equivalent to constructing dense weight matrices containing null spaces in the directions of the biases in the parameter space. Thus, $\mathbf {C}_k^{+}$ is indeed a pseudo-inverse. Table 3 indicates how SIVW is applied with respect to various data types across the core, lithospheric, and tidal parameter subspaces. The remaining parameter subspaces are influenced by all data and have only a “nominal” version.

**Table 3:** CIY4 SIVW application, where the “x” indicate the QD latitude and sun position of the data type and which parameters it directly influences

Type	QD latitude			Sun position		Nominal			Nuisance		
	Low	Mid	High	Light	Dark	Core	Lithosphere	Tide	Core	Lithosphere	Tide
OHM-*NEC*	x	x	x		x	x	x	x			
OHM-*NEC*	x	x	x	x					x	x	x
Single-*F*	x	x	x		x	x	x	x			
Single-*BP*3	x	x			x	x	x	x			
Diffs-*F*	x	x	x		x	x	x	x			
Diffs-*NEC*	x	x			x	x	x	x			
Diffs-*F*	x			x					x	x	x
Diffs-*NEC*	x			x					x	x	x
Diffs-*F*		x	x	x			x	x	x		
Diffs-*NEC*		x		x			x	x	x		
Sums-*F*	x	x	x		x	x				x	x
Sums-*NEC*	x	x			x	x				x	x
Sums-*F*	x	x	x	x					x	x	x
Sums-*NEC*	x	x		x					x	x	x

#### Constraints

For the CIY4 model, the number of explicit quadratic constraints minimized is $N_q=8$ in Eq. 8, although the linear equality constraints can also be expressed this way. They are distributed as five distinct smoothing constraints, i.e., $\mathbf {x}_j^{\prime }=\mathbf {0}$, on the core and lithospheric fields, which includes the mean squared second and third time derivatives of the radial component of the magnetic field, $B_r$, at the core–mantle Boundary (CMB) at $3480\,\hbox {km}$ radius over the entire time domain of the model, denoted as “$\mathcal{P}\langle |{\ddot{B}_r}|^2\rangle$” and “$\mathcal{P}\langle |{\dddot{B}_r}|^2\rangle$,” respectively, and additional customized smoothing of $\dddot{B}_r$ applied to the $(n=1,m=0)$ and $(n=2,m=0)$ harmonics, denoted as “$\mathcal{P}\langle {\dddot{B}_{r,n=1,m=0}^2}\rangle$” and “$\mathcal{P}\langle {\dddot{B}_{r,n=2,m=0}^2}\rangle$,” respectively. The inclusion of smoothing the third time derivative of the core field, with special treatment of the zonal harmonics, follows an approach previously applied with success in the CHAOS model series in order to study field accelerations (see Olsen et al. 2014; Finlay et al. 2016). The smoothing applied to the second time derivative is rather weak, and note the difference compared to the CHAOS model series, where the constraint on the second time derivative is applied only at the model end points whereas it is applied across the entire time domain here. The high-degree lithosphere ($n\ge 85$) is smoothed by minimizing the mean square $B_r$ component over Earth’s mean surface at $6371.2\,\hbox {km}$ and is denoted as “$\mathcal{P}\langle \left| {\mathbf {B}_{n\ge 85}}\right| ^2\rangle$.”

Following Sabaka et al. (2004, 2015), the ionospheric field is smoothed using two constraints, where the first minimizes nightside *E*-region currents, denoted as “$\mathcal{P}\langle \left| {\mathbf {J}_{\mathrm{eq},\mathrm{MLT}:21{-}05}}\right| _2^2\rangle$,” which measures the mean square magnitude of the *E*-region equivalent currents ${\mathbf {J}}_{\mathrm{eq}}$ flowing at $110\,\hbox {km}$ altitude over the nighttime sector, defined as magnetic local time (MLT) $21{:}00{-}05{:}00$ hours, through the year. The second, denoted as “$\mathcal{P}\langle \left| {\nabla _s^2\mathbf {J}_{\mathrm{eq},p>0,\mathrm{mid{-}lat}}}\right| _2^2\rangle$,” measures the mean square magnitude of the surface Laplacian of the diurnally varying portion of ${\mathbf {J}}_{\mathrm{eq}}$ at mid-latitudes at all local times.

As for the magnetospheric and associated induced fields, their solution stability is heavily dependent on the data distribution during each 1 hr bin. Because there are so many bins, an automated procedure was developed in Sabaka et al. (2015) in which the Euclidean ($\ell _2$) length of the magnetospheric/induced coefficients in each bin is minimized and is denoted as “${\mathcal{P}}\langle \left| {{\mathbf {p}}_{\mathrm{mag/ind}}}\right| _2^2\rangle$.” The effect is to add a scalar multiple of the identity matrix, $\lambda \mathbf {I}$, to the normal matrix corresponding to these parameters, $\mathbf {E}_{mi}$. This damping parameter is then determined by first solving

13 $$\begin{aligned} \left( {\mathbf {E}_{mi}+\lambda \mathbf {I}}\right) \mathbf {s}=\left( {\mathbf {E}_{mi}+\lambda \mathbf {I}}\right) \mathbf {1}, \end{aligned}$$

where $\mathbf {1}$ is a vector of ones, and then increasing $\lambda$ from zero until

14 $$\begin{aligned} \left| {\mathbf {1}-\mathbf {s}}\right| _{\infty } < 10^{-8}, \end{aligned}$$

is satisfied, where $\left| {\cdot }\right| _{\infty }$ is the $\ell _{\infty }$ norm. Further details of the algorithm may be found in Sabaka et al. (2015).

Because the field induced by the magnetosphere is represented as a degree one internal potential field with 1 hour bin discretization in time, it should be clear that it can describe the same signal as the core temporal basis and thus represents a co-linearity that cannot be uniquely resolved. Sabaka and Olsen (2006) and Sabaka et al. (2013) developed a set of linear equality constraints that were applied in Sabaka et al. (2015) that force each induced SH time series to be orthogonal to each core SV temporal basis function, including the constant, through time. This results in an induced field that is high-frequency in nature, but is sufficient for what is expected to be encountered. These constraints are manifested in Eq. 8 via the $\mathbf {G}$ matrix whose description may be found in Sabaka and Olsen (2006) and Sabaka et al. (2013). As mentioned earlier, the linear equality constraints can be expressed as quadratic constraints, denoted as $\mathcal{P}\langle \left| {\mathbf {p}_{\mathrm{ind}\perp \mathrm{core}}}\right| _2^2\rangle$, in which case the associated damping parameter $\lambda \rightarrow \infty$. Table 4 shows the values of the damping parameters associated with the various quadratic constrains used in CIY4.

**Table 4:** CIY4 damping parameter values

Norm	Damping parameter ($\lambda$)	
*Core*		
$\mathcal{P}\langle {\dddot{B}_r^2}\rangle$	$1.0\times 10^1\,({\mathrm{nT}}\cdot {\mathrm{year}}^{-3})^{-2}$	
$\mathcal{P}\langle {\dddot{B}_{r,n=1,m=0}^2}\rangle$	$3.0\times 10^2\,(\mathrm{nT}\cdot \mathrm{year}^{-3})^{-2}$	
$\mathcal{P}\langle {\dddot{B}_{r,n=2,m=0}^2}\rangle$	$1.0\times 10^1\,(\mathrm{nT}\cdot \mathrm{year}^{-3})^{-2}$	
$\mathcal{P}\langle {\ddot{B}_r^2}\rangle$	$4.0\times 10^{-2}\,(\mathrm{nT}\cdot \mathrm{year}^{-2})^{-2}$	
*Lithosphere*		
$\mathcal{P}\langle \left| {\mathbf {B}_{n\ge 85}}\right| ^2\rangle$	$2.0\times 10^1\,(\mathrm{nT})^{-2}$	
*Ionosphere*		
$\mathcal{P}\langle \left| {\mathbf {J}_{\mathrm{eq},\mathrm{MLT}:21{-}05}}\right| _2^2\rangle$	$4.0\times 10^7\,(\mathrm{A}\cdot \mathrm{km}^{-1})^{-2}$	
$\mathcal{P}\langle \left| {\nabla _s^2\mathbf {J}_{\mathrm{eq},p>0,\mathrm{mid{-}lat}}}\right| _2^2\rangle$	$1.0\times 10^0\,(\mathrm{A}\cdot \mathrm{km}^{-3})^{-2}$	
*Magnetosphere/induced*		
${\mathcal{P}}\langle \left| {\mathbf{p}}_{\mathrm{mag/ind}} \right|_{2}^{2}\rangle$	Variable $(\mathrm{nT})^{-2}$	
$\mathcal{P}\langle \left| {\mathbf {p}_{\mathrm{ind}\perp \mathrm{core}}}\right| _2^2\rangle$	$\infty \,(\mathrm{nT})^{-2}$	

## Results and discussion

### Residual statistics

The weighted residual statistics for the CIY4 model are shown for the field and the NS sums and differences of the *Alpha* and *Bravo* satellites in Table 5 and continued in Table 6 for the *Charlie* satellite, the EW sums and differences between *Alpha* and *Charlie*, and the field of the OHMs. For the satellite data, the categories reflect the SIVW application scheme. Weighted statistics are shown because these are more representative of how the estimator treats the data types in the IRLS framework. The weighted means and root-mean-squares, $\mu _w$ and $r_w$, respectively, are related to the Huber weights in Eq. 12 as

15 $$\begin{aligned} \mu _w= & {} \sum _i^K w_i e_i/\sum _i w_i, \end{aligned}$$

16 $$\begin{aligned} r_w^2= & {} \sum _i^K w_i e_i^2/\sum _i w_i, \end{aligned}$$

where *K* is the number of measurements and $e_i$ and $w_i$ are the *i*th residual and Huber weight for a particular component, respectively, at the final iterate.

*Alpha* and *Charlie* show very similar residual statistics as expected since they constitute the low satellite pair, while *Bravo* shows slightly higher residuals. The expected properties of larger residuals at higher QD latitudes and on the light versus nightside appear to hold. The differences tend to exhibit the best fits while the sums are the worst of all the measurement types, particularly the light-side sums in the *E* component at low QD latitudes, probably due to radial currents (toroidal magnetic field) connected to the equatorial electrojet, and *N* and *E* components at mid QD latitudes, which is likely due to field-aligned currents. The EW residual differences also appear to be somewhat larger than in the NS direction, but this will at least be partly due to the differencing of two separate instruments that have slightly different biases. Although the *B* and *F* field components are in slightly different directions, i.e., in the computed and observed field directions, respectively, their residual statistics for a given satellite are very similar, as one would expect. The OHMs also exhibit the same property of larger residuals at high QD latitudes and during sunlit conditions.

**Table 5:** CIY4 weighted residual statistics

Origin	Type	Sun	Comp	QD latitude								
				Low			Mid			High		
				*K*	$\mu _w$	$r_w$	*K*	$\mu _w$	$r_w$	*K*	$\mu _w$	$r_w$
Alpha	Field	Dark	*B*	262,005	− 0.664	1.834	1,176,307	0.034	1.506			
			*P*	262,005	0.193	1.914	1,176,307	− 0.025	2.511			
			3	262,005	0.038	1.889	1,176,307	0.094	2.445			
			*F*	262,005	− 0.645	1.825	1,176,307	0.045	1.497	710,017	− 0.075	5.553
	NS differences	Dark	*N*	130,908	0.002	0.201	587,671	0.003	0.313			
			*E*	130,908	0.001	0.334	587,671	0.000	0.360			
			*C*	130,908	− 0.011	0.335	587,671	0.001	0.257			
			*F*	130,865	0.007	0.163	587,645	− 0.010	0.179	354,475	− 0.023	0.937
		Light	*N*	165,351	− 0.004	0.760	807,365	− 0.006	0.555			
			*E*	165,351	0.002	0.807	807,365	− 0.001	0.839			
			*C*	165,351	− 0.007	0.917	807,365	0.004	0.520			
			*F*	165,349	− 0.002	0.651	807,240	0.010	0.318	683,385	− 0.037	1.095
	NS sums	Dark	*N*	130,908	− 1.175	3.165	587,671	− 0.714	3.770			
			*E*	130,908	0.270	3.194	587,671	0.018	4.069			
			*C*	130,908	0.016	3.110	587,671	0.105	2.936			
			*F*	130,865	− 1.016	3.035	587,645	− 0.010	2.520	354,475	− 0.101	8.919
		Light	*N*	165,351	2.154	7.117	807,365	− 0.664	6.885			
			*E*	165,351	− 0.232	12.185	807,365	0.162	9.156			
			*C*	165,351	0.330	7.337	807,365	− 0.005	5.360			
			*F*	165,349	2.113	6.798	807,240	− 0.647	5.039	683,385	− 3.831	12.903
Bravo	Field	Dark	*B*	259,916	− 0.792	3.013	1,169,539	0.071	2.227			
			*P*	259,916	0.262	2.425	1,169,539	0.086	2.893			
			3	259,916	− 0.131	1.999	1,169,539	0.067	3.231			
			*F*	259,916	− 0.819	3.024	1,169,539	0.081	2.224	715,893	0.086	5.437
	NS differences	Dark	*N*	129,850	0.005	0.195	584,275	0.006	0.316			
			*E*	129,850	− 0.002	0.325	584,275	− 0.002	0.364			
			*C*	129,850	− 0.002	0.330	584,275	0.001	0.256			
			*F*	129,820	0.011	0.164	584,270	− 0.009	0.196	357,613	− 0.023	0.850
		Light	*N*	163,615	− 0.002	0.671	795,240	− 0.000	0.540			
			*E*	163,615	0.004	0.741	795,240	− 0.002	0.822			
			*C*	163,615	0.005	0.838	795,240	0.001	0.502			
			*F*	163,592	− 0.001	0.565	795,286	0.009	0.303	673,198	− 0.035	0.992
	NS sums	Dark	*N*	129,850	− 1.211	5.068	584,275	− 0.792	5.476			
			*E*	129,850	0.366	3.972	584,275	0.232	4.702			
			*C*	129,850	− 0.220	3.104	584,275	0.016	3.406			
			*F*	129,820	− 1.173	4.931	584,270	0.162	3.650	357,613	0.122	8.862
		Light	*N*	163,615	2.341	8.171	795,,240	− 0.241	7.909			
			*E*	163,615	− 0.506	12.955	795,,240	− 0.253	9.850			
			*C*	163,615	0.093	7.026	795,,240	− 0.121	5.684			
			*F*	163,592	2.199	7.662	795,286	− 0.654	5.681	673,198	− 3.838	12.618

The $\mu _w$ and $r_w$ are in units of *nT*

**Table 6:** CIY4 weighted residual statistics *continued*

Origin	Type	Sun	Comp	QD latitude								
				Low			Mid			High		
				*K*	$\mu _w$	$r_w$	*K*	$\mu _w$	$r_w$	*K*	$\mu _w$	$r_w$
Charlie	Field	Dark	*B*	259,643	− 0.586	1.846	1,167,024	0.100	1.604			
			*P*	259,643	0.110	1.912	1,167,024	− 0.101	2.578			
			3	259,643	− 0.159	2.241	1,167,024	0.076	2.662			
			*F*	259,643	− 0.604	1.851	1,167,024	0.097	1.604	705,882	0.002	5.599
	NS differences	Dark	*N*	129,773	0.003	0.213	582,975	0.002	0.332			
			*E*	129,773	− 0.001	0.342	582,975	− 0.001	0.372			
			*C*	129,773	− 0.006	0.355	582,975	− 0.001	0.278			
			*F*	129,723	0.006	0.174	582,999	− 0.012	0.186	352,487	− 0.024	0.941
		Light	*N*	164,450	− 0.004	0.764	802,108	− 0.006	0.571			
			*E*	164,450	− 0.006	0.811	802,108	− 0.004	0.848			
			*C*	164,450	− 0.001	0.926	802,108	0.001	0.533			
			*F*	164,346	0.000	0.652	802,180	0.009	0.320	678,530	− 0.037	1.096
	NS sums	Dark	*N*	129,773	− 1.033	3.141	582,975	− 0.577	3.965			
			*E*	129,773	0.172	3.198	582,975	− 0.107	4.147			
			*C*	129,773	− 0.312	3.569	582,975	0.086	3.299			
			*F*	129,723	− 0.940	3.049	582,999	0.204	2.662	352,487	0.028	8.983
		Light	*N*	164,450	2.129	7.095	802,108	− 0.682	7.054			
			*E*	164,450	− 0.502	11.935	802,108	0.045	9.276			
			*C*	164,450	0.104	7.662	802,108	− 0.020	5.562			
			*F*	164,346	2.108	6.780	802,180	− 0.659	5.051	678,530	− 3.739	12.857
Alpha/Charlie	EW differences	Dark	*N*	234,862	0.108	0.407	1,057,587	0.081	0.495			
			*E*	234,862	0.015	0.905	1,057,587	0.009	0.910			
			*C*	234,862	− 0.122	0.617	1,057,587	0.014	0.427			
			*F*	235,259	− 0.095	0.373	1,060,137	− 0.069	0.341	654,422	− 0.065	0.555
		Light	*N*	297,697	− 0.033	0.648	1,451,527	− 0.027	0.788			
			*E*	297,697	− 0.023	2.146	1,451,527	− 0.009	1.862			
			*C*	297,697	− 0.069	1.285	1,451,527	0.018	0.746			
			*F*	298,285	0.028	0.548	1,453,701	0.008	0.491	1,218,886	− 0.072	0.616
	EW sums	Dark	*N*	234,862	− 1.175	3.086	1,057,587	− 0.677	3.703			
			*E*	234,862	0.280	2.984	1,057,587	− 0.050	4.007			
			*C*	234,862	− 0.262	2.822	1,057,587	0.096	2.797			
			*F*	235,259	− 1.063	2.982	1,060,137	0.168	2.495	654,422	− 0.005	9.166
		Light	*N*	297,697	2.270	7.029	1,451,527	− 0.630	6.772			
			*E*	297,697	− 0.367	11.680	1,451,527	0.057	8.920			
			*C*	297,697	0.099	7.038	1,451,527	− 0.057	5.194			
			*F*	298,285	2.256	6.720	1,453,701	− 0.566	4.945	1,218,886	− 3.858	13.066
OHM	Field	Dark	*N*	30,040	− 0.001	4.422	363,714	− 0.004	4.133	103,441	0.009	14.686
			*E*	30,040	0.000	5.246	363,714	0.002	4.847	103,441	0.000	11.599
			*C*	30,040	0.001	4.499	363,714	0.003	3.675	103,441	0.005	14.850
		Light	*N*	38,022	2.688	10.602	533,305	0.631	6.861	222,753	3.384	18.633
			*E*	38,022	− 1.961	9.344	533,305	− 1.750	7.882	222,753	− 3.384	15.666
			*C*	38,022	− 0.073	9.957	533,305	− 0.180	5.452	222,753	− 0.927	17.620

The $\mu _w$ and $r_w$ are in units of *nT*

**Fig. 2:**
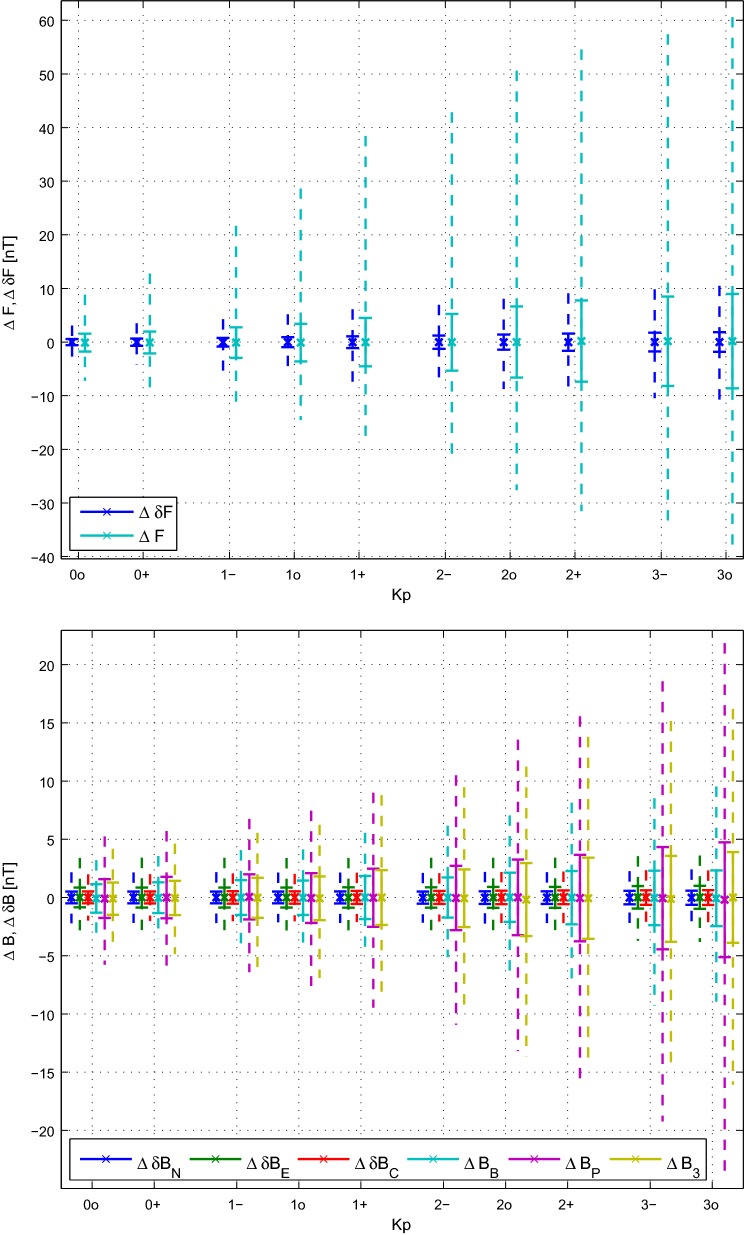
Residuals of the scalar field $\Delta F$ and differences $\Delta \delta F$ (top) and the vector field $\Delta \mathbf {B}$ and differences $\Delta \delta \mathbf {B}$ (bottom) from CIY4 for *Swarm Alpha* with respect to *Kp* activity level. The “X” denote the weighted means while the bars indicate weighted standard deviations. The dashed lines indicate $\pm\, 99\%$ of the residual ranges. Note that the vector components for the field data are $\textit{BP3}$, but are $\textit{NEC}$ for the difference data

As mentioned in section “Data selection,” the *Swarm* vector data are chosen during times when $Kp \le 3^0$ and so it is interesting to see the effect of this activity level on how well the data are fit. To this end Fig. 2 shows the field and difference residuals for the scalar and vector components of *Alpha* measurements as a function of *Kp* activity level. The residuals of the *Bravo* and *Charlie* satellites show patterns similar to that of *Alpha* and so are not included. One can see diminished ranges of scalar difference residuals compared to vector field residuals, as the former increase slightly and the latter more profoundly with *Kp* level. The ranges of the vector differences in the $\textit{NEC}$ frame are much smaller than the field in the $\textit{BP3}$ frame with the former appearing to be almost invariant to *Kp* activity level in contrast to the latter, which also increase with *Kp* level. This is intriguing since it means that more liberal bounds may be placed on *Kp* selection levels for differences (as previously reported by Olsen et al. 2016), allowing for better data coverage. Finally, the scalar residuals appear to increase asymmetrically (skewed toward more positive values) with increasing *Kp* as opposed to the symmetric increase seen in vector residuals, which is due to the presence of high latitude scalar data.

### Parameter separability

The main advantage of the CI algorithm is that it co-estimates the parameters from all considered sources in order to obtain optimal separation. However, one must be aware of co-linearities between the parameters that can amplify noise in the measurements and contaminate the estimate. Though this may appear to be a weakness in the co-estimation compared to the sequential approach, these co-linearities may be present in both, but are only readily detectable in the former. The co-linearities may be measured by inspecting the classic correlation coefficient, $\rho$, between parameter pairs.

Because the CI algorithm initially removes the magnetospheric and associated induced parameters through Gaussian elimination, only $\rho$ between non-magnetospheric/induced parameters is directly available. Of these parameters, there are five groups with $\left| {\rho }\right| \ge 0.7$ inter-correlations. The first group is between spline parameters of the core/SV basis functions that are mostly positive such that many are $\rho \approx 1$. This was also detected in CM5 (Sabaka et al. 2015) and is due to the influence of the core/SV quadratic constraints that contain null spaces. The second group is between certain zonal pairs of coefficients in the nuisance crustal field of the form $g_n^0$ and $g_{n+2}^0$ that are positive and can reach 0.74. The third group is between ionospheric parameters which are mostly negative and can be as low as $-\,0.96$. These were not detected in CM5, but are also due to the influence of the quadratic constraints applied to the ionosphere that have large ranges in their eigenvalues. This suggests that these constraints are relatively stronger in CIY4 compared to CM5. The fourth group is between OHM biases whose locations are in close proximity such that some $\rho \approx 1$. This was also seen in CM5 and is due to the similarity of the crustal field at the two locations, which is discussed in more detail in Sabaka et al. (2015). The last group concerns the Euler alignment angles and are either negative correlations between the *x*- and *z*-axis rotations for the same satellite in a given bin that can reach $-\,0.96$ due to the intermediate rotation of approximately $76^{\circ }$ about the *y*-axis or positive correlations between similar rotation axes in adjacent bins between $\textit{Alpha}$ and $\textit{Charlie}$ that can reach 0.98. The first and third groups are the result of smoothing constraints and are shown in Appendix A of Sabaka et al. (2015) to not adversely affect the solution and despite some large positive and negative $\rho$ values in last group, it appears, as in CM5, that there are no deleterious effects.

**Fig. 3:**
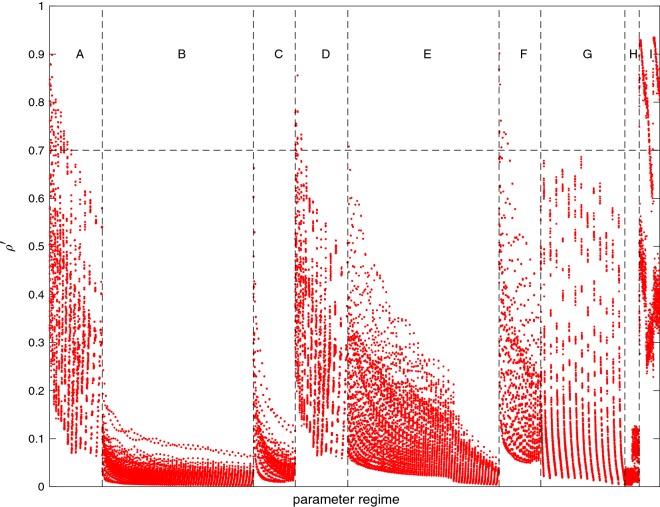
The $\rho ^{\prime }$ value for each non-magnetospheric/induced parameter. The horizontal dashed line at 0.7 represents the geometric “half-way point” between uncorrelated and perfectly correlated. The vertical dashed lines delineate between different parameter regimes: (A) nominal core/SV, (B) nominal crust, (C) nominal $M_2$ tide, (D) nuisance core/SV, (E) nuisance crust, (F) nuisance $M_2$ tide, (G) ionosphere, (H) OHM biases, and (I) *Swarm* alignment Euler angles

As for correlations between the magnetospheric/induced parameters and the others, the parameter subspace correlation coefficient, $\rho ^{\prime }$, introduced in Sabaka et al. (2015), may be used to bound $\rho _{ij}$ between the *i*th magnetospheric/induced parameter and another *j*th parameter of interest such that $\left| {\rho _{ij}}\right| \le \rho ^{\prime }_j$ for all *i*. Figure 3 shows $\rho ^{\prime }$ for all non-magnetospheric/induced parameters where the letters indicate the parameter regime. Most $\rho ^{\prime }$ are well below the $\rho =0.7$ threshold and all $\rho ^{\prime }<0.94$. It peaks above 0.7 for nominal and nuisance core/SV and nuisance $M_2$ tidal parameters and several Euler angles. The pattern is generally similar to that seen in CM5 and the correlations with the magnetospheric/induced parameters do not appear to be detrimental.

### Core field

**Fig. 4:**
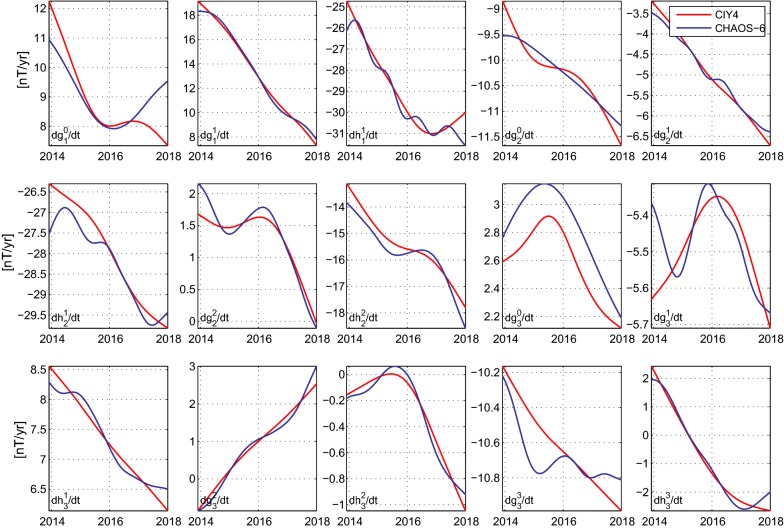
The CIY4 (red) and CHAOS-6-x5 (blue) SV coefficients from 2014 to 2018 for SH degrees $n=1{-}3$

Figure 4 presents a comparison between the lowest SHs of the SV of the core field part of the CIY4 model and the same quantities from the latest update of the CHAOS-6 model (Finlay et al. 2016), CHAOS-6-x5. The latter were also derived using *Swarm* data (L1b data product version 0503) up to the end of 2017 but were in addition constrained by annual differences of ground observatory monthly means. Despite the differences in their data selection and modeling techniques, the time dependence of the CIY4 and CHAOS-6-x5 SV coefficients between 2014 and 2018 are in good agreement. In particular, the almost linear slopes (corresponding to a constant secular acceleration (SA)) in the coefficients $\dot{g^1_1}$, $\dot{h^2_2}$ and $\dot{g^2_3}$ match very well, while changes in the slope of the SV, corresponding to pulses in SA, are observed at similar times in both models in the time series of $\dot{g^0_1}$, $\dot{g^2_2}$, $\dot{g^0_3}$, $\dot{g^1_3}$. Overall, the time dependence of the SV in CIY4 is simpler than that in CHAOS-6-x5, and there are some differences in the starting levels since CHAOS-6-x5 contains other data sources at earlier times, but the major features are shared by the two models.

**Fig. 5:**
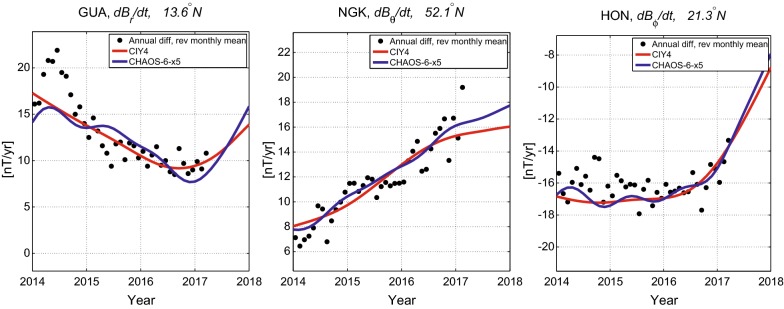
Comparison of CIY4 (red) and CHAOS-6-x5 (blue) fits to ground observatory SV data (annual differences of revised monthly means) in the $\dot{B}_r$, $\dot{B}_{\theta }$, and $\dot{B}_{\phi }$ components of GUA, NGK, and HON, respectively

Example comparisons of the SV predicted at ground observatories, between 2014 and 2018, by CIY4 and CHAOS-6-x5 are shown in Fig. 5. Once more the trends predicted by the two models, including changes in the slope of the SV, are in close agreement and more importantly they also describe well the SV signal seen in annual differences of the ground observatory monthly means, especially on timescales longer than a year. This is particularly impressive for CIY4 since (unlike CHAOS-6) it is not asked to directly fit annual differences of monthly mean observatory data. Both models show an interesting change in the slope of the SV in the Pacific region in late 2016/early 2017, for example, in $\dot{B_r}$ at GUA and in the $\dot{B}_{\phi }$ at HON (see Fig. 5). A change in slope is also clearly seen at this time in the annual differences of monthly means in $\dot{B}_{\phi }$ at HON. This may possibly be a signature of a geomagnetic jerk type event taking place in the Pacific region; CIY4 is clearly able to follow such events.

**Fig. 6:**
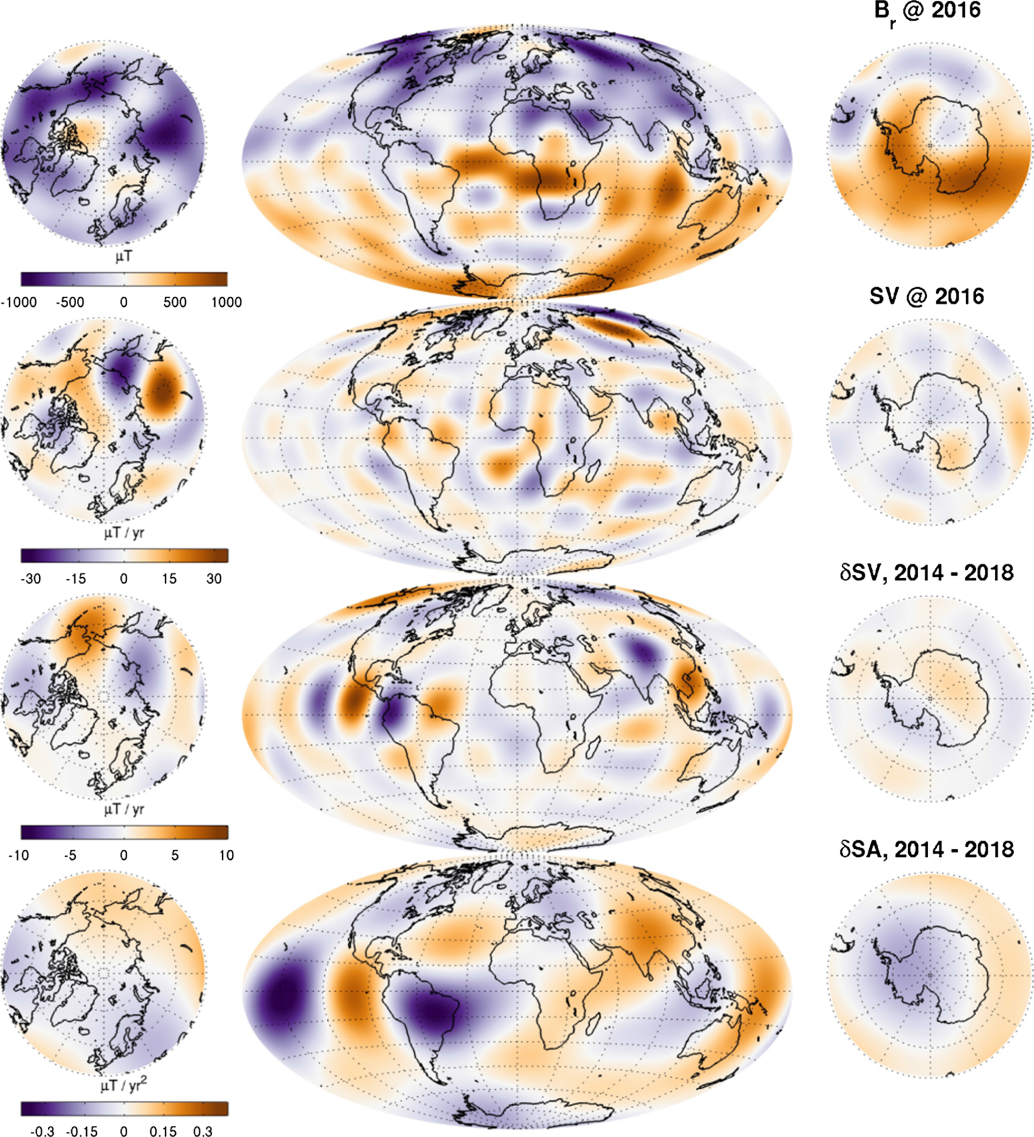
From the top, maps of the CIY4 radial field and SV at 2016, the change in radial SV from 2014 to 2018, and the change in radial SA from 2014 to 2018, at the CMB, truncated at SH degree 13

Further details concerning the structure of the core field and its time changes at the outer edge of the geodynamo (i.e., the CMB) are given in Fig. 6. For consistency, all plots in Fig. 6 are truncated at SH degree 13; the time-dependent internal field from CIY4 is stable at the CMB up to this degree. The CMB radial field and SV shown in the top two panels display familiar structures, with intense flux patches at high latitudes (under Siberia and Canada and under Antarctica toward South America and Australia), and at low latitudes under the hemisphere centered under the Atlantic, and with reversed flux features visible in the Southern Atlantic. The radial field SV is largest at low latitudes under the hemisphere centered on the Atlantic and in the northern hemisphere under Canada and Siberia. In contrast the Pacific and the Southern polar region are quiet.

Considering the SA and its time changes in CIY4 provides a number of new insights concerning the latest changes in the core field. The third panel in Fig. 6 shows the estimated change in the radial SV (i.e., the accumulated radial SA) at the CMB over the first 4 years of the *Swarm* mission, between 2014 and 2018. With 4 years of data it is now possible to confirm that using *Swarm* data alone results in similar SA patterns to those inferred previously from CHAMP-only and mixed CHAMP-*Swarm* field models (Chulliat and Maus 2014; Finlay et al. 2015). In particular, between 2014 and 2018 significant field acceleration at the core surface has occurred (i) at low latitudes under northern South America and extending into the Eastern Pacific, (ii) under South-East Asia, and (iii) under Alaska and Siberia. The latter point confirms that CIY4 shows similar patterns of field accelerations at high northern latitudes to those highlighted by Livermore et al. (2017), indicating these features are not a consequence of the gap between CHAMP and *Swarm* or due to any differences in the observing capabilities of these missions. Localized changes in CMB field acceleration patterns have previously been linked to the occurrence of geomagnetic jerks (Olsen and Mandea 2007; Chulliat et al. 2010). The largest differences in the CIY4 radial field SA between 2014 and 2018 are found under northern South America and under the equatorial Pacific, consistent with a possible jerk-like features found toward the end of the most up-to-date ground observatory SV series from this region (see Fig. 5). The prospect of detailed magnetic field observations from the *Swarm* constellation during a jerk event is tantalizing, but a detailed assessment needs to await the accumulation of longer ground observatory series. It is in any case striking that large changes in field acceleration occur in the Pacific hemisphere, despite the lower amplitude of secular variation in this region.

### Lithospheric field

Following Sabaka et al. (2013), the CIY4 lithospheric field is compared to that of the LCS-1 (Olsen et al. 2017) and MF7 (Maus 2010) models using three metrics, the first being the Lowes-Mauersberger spectrum, $R_n(r)$, of Lowes (1966) defined as

17 $$\begin{aligned} R_n(r) & = (n+1)\left( {a\over r}\right) ^{2n+4} \sum _{m=0}^n \left[ {\left( {g_n^m}\right) ^2+\left( {h_n^m}\right) ^2}\right] , \\ & = (n+1)\left( {a\over r}\right) ^{2n+4} \sum _{m=0}^n \left| {\gamma _n^m}\right| ^2, \end{aligned}$$

where *a* and *r* are the reference and evaluation radii, respectively, $\gamma _n^m$ are the complex, and $g_n^m$ and $h_n^m$ are the real Gauss coefficients of the SH expansion. The second metric is the degree correlation between two models

18 $$\begin{aligned} \rho _n={\sum _{m=0}^n \left[ {g_{n,1}^m g_{n,2}^m+h_{n,1}^m h_{n,2}^m}\right] \over \sqrt{\sum _{m=0}^n \left[ {\left( {g_{n,1}^m}\right) ^2+\left( {h_{n,1}^m}\right) ^2}\right] \sum _{m=0}^n \left[ {\left( {g_{n,2}^m}\right) ^2+\left( {h_{n,w}^m}\right) ^2}\right] }}, \end{aligned}$$

where $g_{n,k}^m$ and $h_{n,k}^m$ are the Gauss coefficients of model “*k*.” The last metric is the matrix of normalized coefficient differences (in %), *S*(*n*, *m*), given by

19 $$\begin{aligned} S(n,m)= \left\{ { \begin{array}{ll} 100 {h_{n,e}^m-h_{n,r}^m\over \sqrt{{1\over 2n+1} \sum _{m=0}^n \left[ {\left( {g_{n,r}^m}\right) ^2+\left( {h_{n,r}^m}\right) ^2}\right] }}, &\quad{} {\mathrm{for}}\; m < 0, \\ 100 {g_{n,e}^m-g_{n,r}^m\over \sqrt{{1\over 2n+1} \sum _{m=0}^n \left[ {\left( {g_{n,r}^m}\right) ^2+\left( {h_{n,r}^m}\right) ^2}\right] }}, &\quad{} {\mathrm{for}}\; m \ge 0, \end{array}}\right. , \end{aligned}$$

where $g_{n,x}^m$ and $h_{n,x}^m$ are the Gauss coefficients of the evaluated and reference models when “*x*” is “*e*” or “*r*,” respectively.

**Fig. 7:**
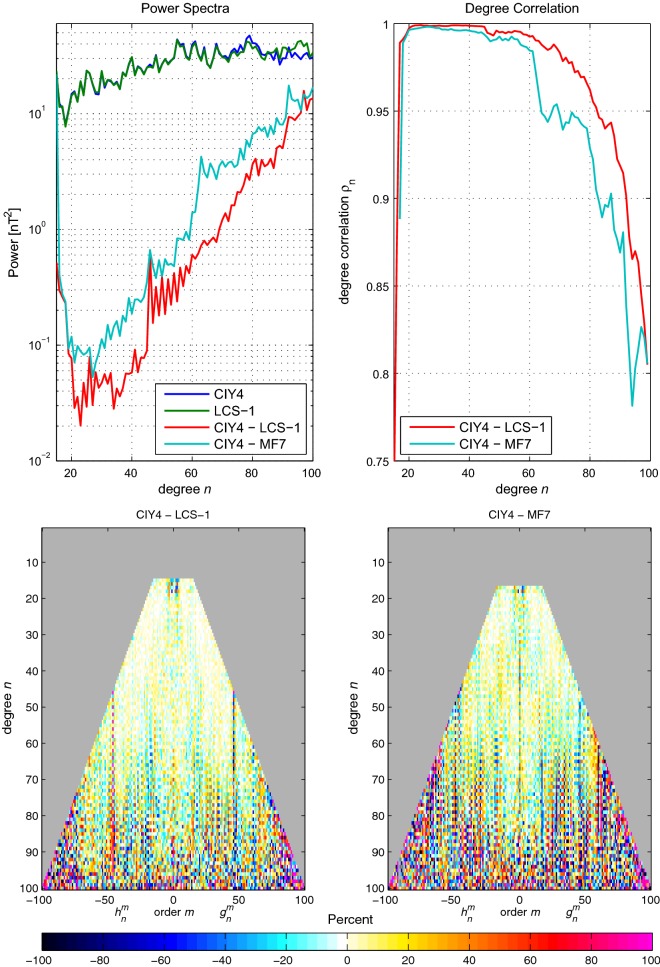
Top left: $R_n(a)$ spectra (Lowes 1966) of CIY4 and LCS-1 and the differences between CIY4 and LCS-1 and between CIY4 and MF7 for $a=6371.2\,\hbox {km}$. Top right: degree correlations $\rho _n$ between CIY4 and LCS-1 and CIY4 and MF7. Bottom: matrices of normalized coefficient differences, *S*(*n*, *m*), of CIY4 with respect to LCS-1 and MF7. All plots are for SH degrees $n=15{-}100$ at epoch 2015.0

Figure 7 shows all three metrics for the lithospheric field defined as degrees $n=15{-}100$ and at epoch 2015.0. The top left panel shows the $R_n(a)$ spectrum at $a=6371.2\,\hbox {km}$ for CIY4 and LCS-1 and for the differences between CIY4 and LCS-1 and MF7. The differences are smaller with respect to LCS-1 than MF7 and all differences are below the actual power over the degree range. Likewise, the top right panel shows degree correlations $\rho _n$ that are higher with respect to LCS-1 than MF7 for all degrees. The correlations are also above 0.8 with respect to LCS-1, which gives confidence that the lithosphere is being extracted well. This is also confirmed in the lower panel where the matrix of normalized coefficient differences shows better agreement between CIY4 and LCS-1 than with MF7, particularly in the sectoral $(n=m)$ terms. It is not surprising that the CIY4 lithosphere agrees better with LCS-1 since both incorporate *Swarm* data.

**Fig. 8:**
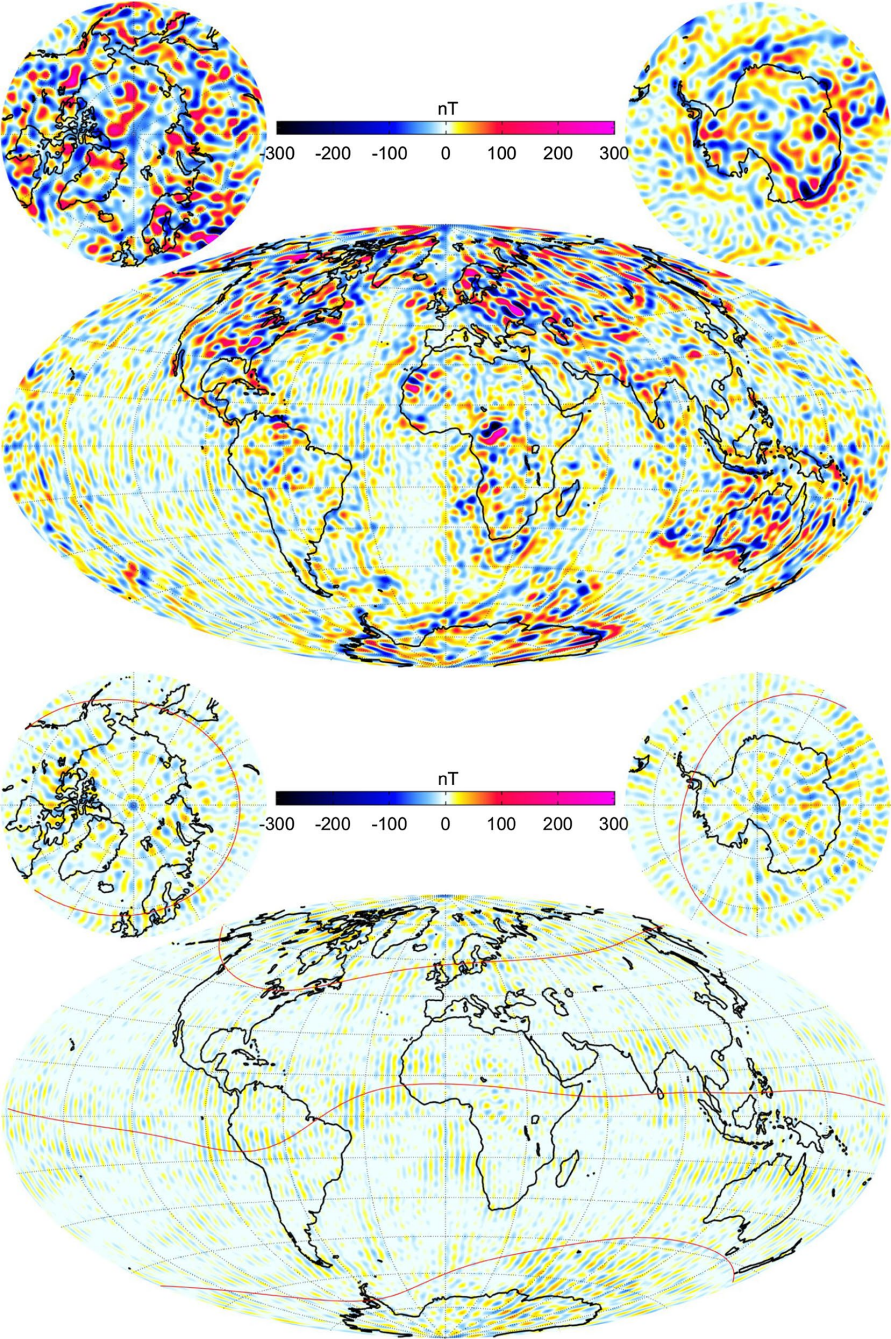
Top: Map of the CIY4 lithospheric field vertical component *Z* at Earth’s surface (ellipsoid WGS84), for SH degrees $n=16{-}100$ at epoch 2015.0. Bottom: difference between CIY4 lithospheric field and the LCS-1 model over the same degree range and at the same epoch as above. Red curves represent QD latitudes of $\pm\, 55^\circ$ and $0^\circ$

A final comparison is shown in Fig. 8 where maps of the *Z* component are plotted and compared on Earth’s ellipsoidal surface (WGS84). The top panel shows the CIY4 lithospheric field for degrees $n=16{-}100$ while the bottom shows the difference between the fields of CIY4 and LCS-1 for the same degree range. Red curves represent the QD latitudes of $\pm\, 55^\circ$ and $0^\circ$ and both maps use the same scale. The models appear to agree well overall with the largest discrepancies in the polar regions, as expected. There also appears to be a faint patchwork of differences in the proximity of low QD latitudes. This may be a result of including dayside differences in determining the nominal lithospheric part of the model. Overall, the quality of the CIY4 lithospheric model is quite encouraging, especially given the altitude of the *Swarm* satellites and the level of magnetic activity compared to the LCS-1 and MF7 models, which include CHAMP data.

### Oceanic $M_2$ tidal field

**Fig. 9:**
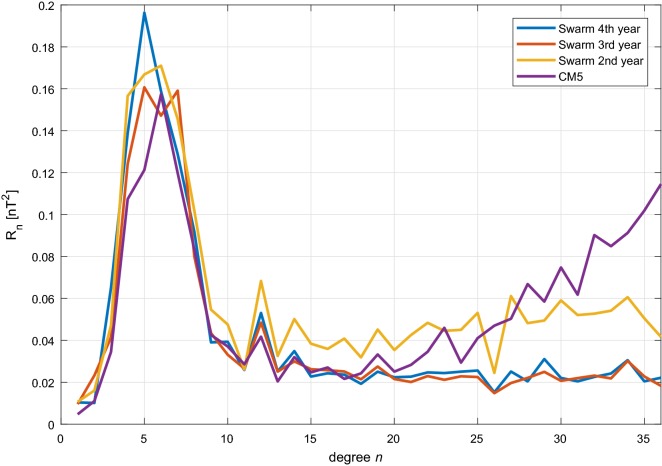
The $R_n(a)$ spectra (Lowes 1966; Sabaka et al. 2015, 2016) of the time-averaged oceanic $M_2$ tidal magnetic field at $a=6371.2\,\hbox {km}$ for SH degrees $n=1{-}36$ from models determined by 2, 3, and 4 (CIY4) years of *Swarm* data and from CHAMP data (CM5)

The oceanic $M_2$ signal was first detected in early CHAMP data by Tyler et al. (2003) and then later in CM5 by Sabaka et al. (2015) and from *Swarm* data in CI1 by Sabaka et al. (2016). Results were in all cases validated by comparison with forward models described by Tyler et al. (2003) and Kuvshinov (2008). Here the progression of models based on increasing amounts of *Swarm* data are compared along with the CM5 results. It is useful to consider the power of the tidal magnetic field using a generalization of the classic $R_n$ spectrum of Lowes (1966) introduced in Sabaka et al. (2015, 2016) and defined as the mean square magnitude of the $M_2$ magnetic field at SH degree *n* over a sphere of radius *r* and over the $M_2$ tidal period given by

20 $$\begin{aligned} R_n\left( {r}\right) =\left( {n+1}\right) \left( {a\over r}\right) ^{2n+4}\left\{ {{1\over 2}\left| {\tau _n^0}\right| ^2+\sum _{m=1}^n\left[ {\left| {\tau _n^m}\right| ^2+\left| {\tau _n^{-m}}\right| ^2}\right] }\right\} , \end{aligned}$$

where $a=6371.2\,\hbox {km}$. The $R_n$ spectra are shown in Fig. 9 for models derived from 2, 3, and 4 (CIY4) years of *Swarm* data and from the entire CHAMP mission (CM5). All models show strong peak regions in the vicinity of degrees $n=4{-}7$ and roughly similar patterns up to about $n=20$. However, at higher degrees the *Swarm* 2nd year model and CM5 diverge with higher power, especially CM5, due no doubt to field contamination. The *Swarm* 3rd and 4th (CIY4) year models show much less power at higher degrees and the latter shows a prominent peak at degree $n=5$. There is a clear reduction in power from the 2nd to 3rd years of *Swarm* data, but not so much between the 3rd and 4th years, which is likely indicating some critical coverage threshold being achieved by the 3rd year or perhaps due to a decrease in solar activity.

**Fig. 10:**
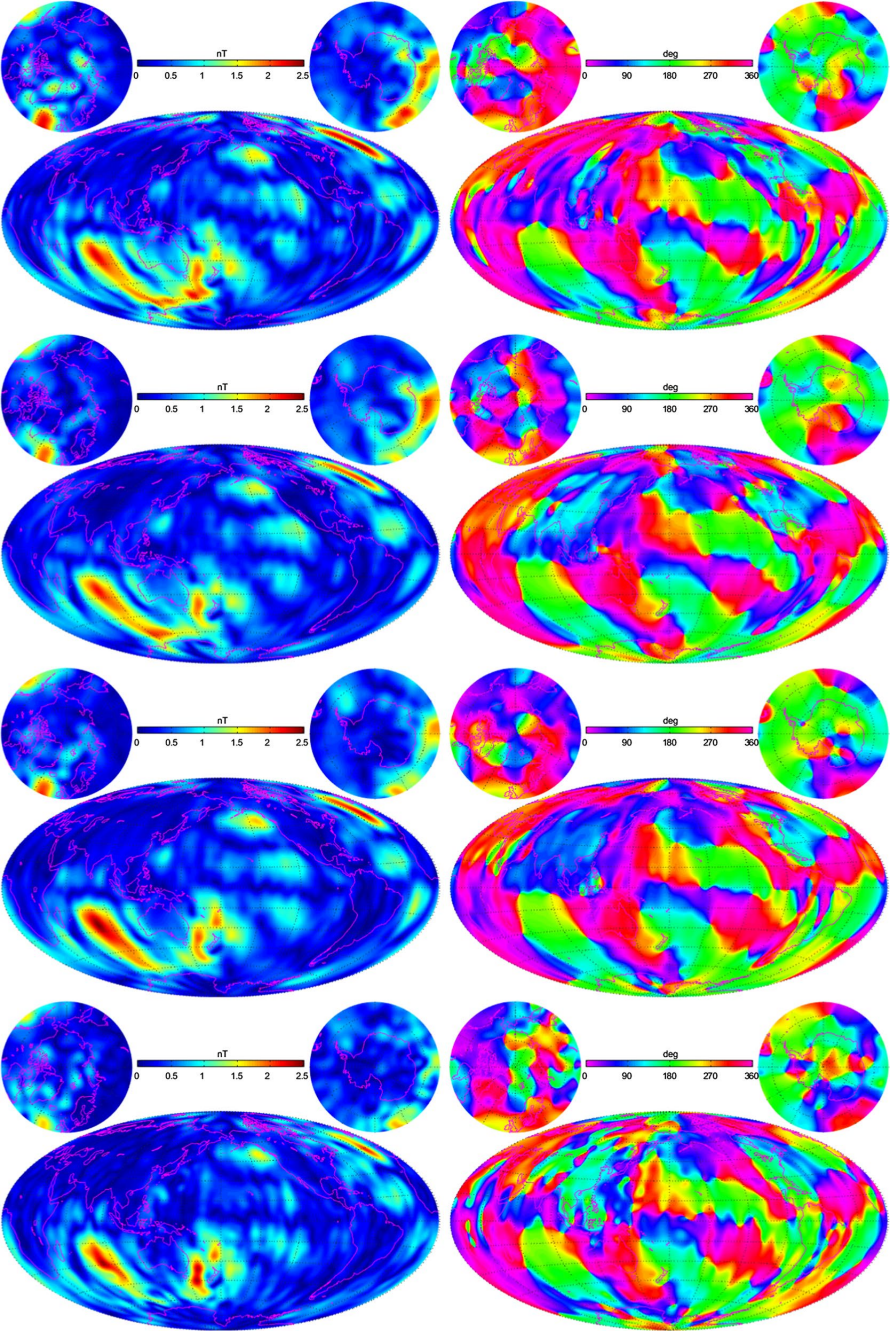
The amplitude (left) and phase (right) of the radial component of the oceanic $M_2$ tidal magnetic field at $430\,\hbox {km}$ altitude as estimated from, starting at the top, 2, 3, and 4 (CIY4) years of *Swarm* data and from CHAMP data (CM5) for SH degrees $n=1{-}36$

As the $M_2$ tide is a periodic phenomenon, it is convenient to decompose its magnetic field in terms of an amplitude and phase, which are indeed shown in Fig. 10 for the radial component at $430\,\hbox {km}$ altitude for SH degrees $n=1{-}36$. From the top of the figure are shown amplitude and phase pairs for the fields derived from *Swarm* data through the 2nd, 3rd, and 4th (CIY4) years of the mission and from the CM5 model at the bottom. The progression confirms what is seen in the power spectra in that small-scale spurious, often north–south trending, features are eliminated as more *Swarm* data are available, culminating in the CIY4 model which is much less noise-prone then the CM5 model derived from CHAMP satellite data. It should be noted, however, that the mid-to-large (and several small) features in the *Swarm* fields appear to be converging to those of CHAMP, thus validating the high-quality measurements of both missions.

**Fig. 11:**
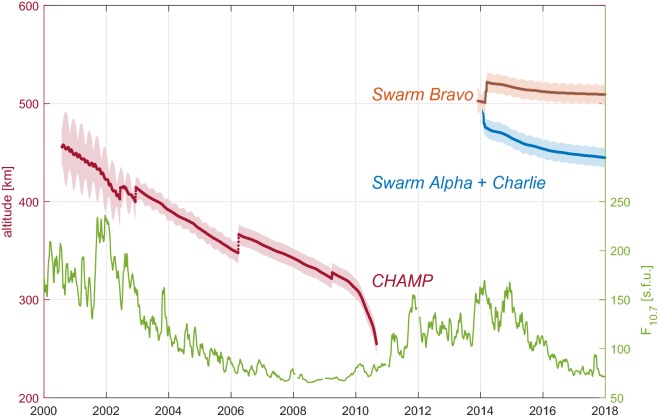
Altitudes of the CHAMP (red) and the *Swarm Bravo* (orange), *Alpha*, and *Charlie* (blue) satellites, as indicated by the left scale, and the $F_{10.7}$ solar radiation index (green), as indicated by the right scale, from 2000 to the end of the CIY4 data envelope. The solid dark lines show the daily mean altitude, while the shaded areas indicate the range (difference between daily max and daily min)

To illustrate the utility of the *Swarm* gradiometric measurements Fig. 11 shows the altitudes of the CHAMP satellite over its mission, the *Swarm* satellites through the end of the CIY4 data envelope, and the $F_{10.7}$ solar radiation index. The CHAMP mission ran for over 10 years during which the final 4 occurred in a period of anomalously low solar activity, hence less magnetic disturbances, and allowed the satellite altitude to go below $350\,\hbox {km}$. In contrast, the *Swarm* mission began flying during a relative high in $F_{10.7}$ for almost 2 years, which has now decreased through the fourth year of the mission. However, until now, the low-pair altitudes have not gone below $450\,\hbox {km}$. Thus, in spite of higher altitudes during relatively longer disturbed times, the *Swarm* constellation has extracted a high quality $M_2$ tidal driven magnetic field.

As stated in the “Introduction”, the CI $M_2$ magnetic field are provided as a new *Swarm* L2 data product. For this it has been decided that $M_2$ SH coefficients will be presented in real notation as opposed to the complex notation used above such that Eq. 6 may be rewritten as

21 $$\begin{aligned} V_{M_2}(\Delta t,\mathbf {r}) &= a \sum _{n=1}^{36} \left( {a\over r}\right) ^{n+1} \sum _{m=0}^n \left[ {\left( {a_n^m \cos {m\phi }+c_n^m \sin {m\phi }}\right) \cos {\omega _{M_2}\Delta t}}\right. \\ &\quad \left.+ {\left( {b_n^m \cos {m\phi }+d_n^m \sin {m\phi }}\right) \sin {\omega _{M_2}\Delta t}}\right] P_n^m(\cos {\theta }), \end{aligned}$$

where the real and complex coefficients are related as

22 $$\begin{aligned} \left\{ { \begin{array}{ll} a_n^0=\mathfrak {R}\left\{ {\tau _n^0}\right\} , &\quad{} b_n^0=-\mathfrak {I}\left\{ {\tau _n^0}\right\} \\ a_n^m=\mathfrak {R}\left\{ {\tau _n^{-m}+\tau _n^m}\right\} , &\quad{} b_n^m=-\mathfrak {I}\left\{ {\tau _n^{-m}+\tau _n^m}\right\} \\ c_n^m=\mathfrak {I}\left\{ {\tau _n^{-m}-\tau _n^m}\right\} , &\quad{} d_n^m=\mathfrak {R}\left\{ {\tau _n^{-m}-\tau _n^m}\right\} \end{array}}\right. , \end{aligned}$$

and the $\mathfrak {I}\left\{ {\cdot }\right\}$ operator takes the imaginary part of the expression only.

### Ionospheric field

**Fig. 12:**
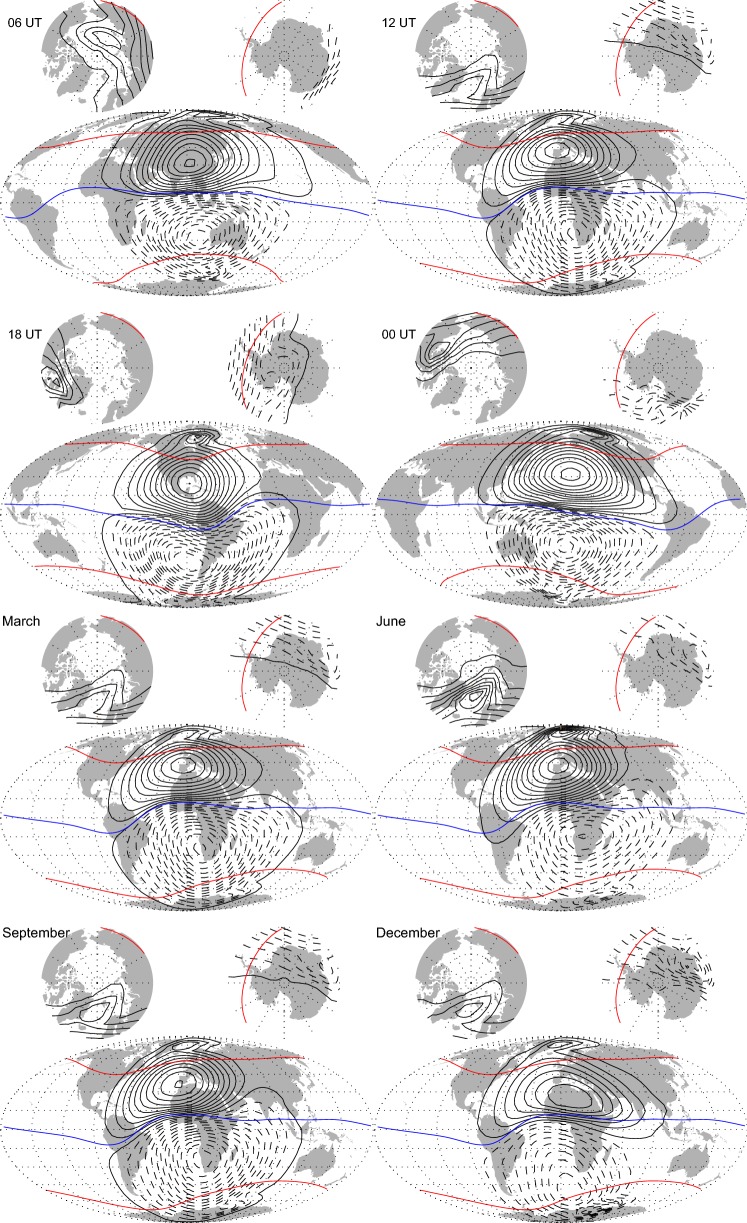
The top four maps show the primary ionospheric *E*-region current function $\Psi$ at $110\,\hbox {km}$ altitude during vernal equinox centered on noon local time for 06, 12, 18, and $00\,\hbox {UT}$. The bottom four maps show $\Psi$ centered at local noon and $12\,\hbox {UT}$ on the 21st day of March, June, September, and December. Solid/dashed contour lines indicate counter-clockwise/clockwise current flow. A $10\,\hbox {kA}$ current flows between contours. Red curves represent QD latitudes of $\pm\, 55^\circ$ and blue represents $0^\circ$. A value of $F_{10.7}=100.0\times 10^{-22}\,\mathrm{W}/\mathrm{m}^2/\mathrm{Hz}$ was used for evaluation

As in Sabaka et al. (2015), the primary ionospheric *E*-region current system is treated as a sheet current at an altitude of $110\,\hbox {km}$ while the secondary system is induced by the primary system via the “1D+oceans” conductivity structure described in section “Ionospheric field.” Figure 12 shows the variability of the equivalent current, i.e., stream, function corresponding to the primary system in two aspects: variation with respect to local time during vernal equinox in the top four maps, and variation with respect to season in the bottom four maps. As the basis functions for the ionosphere in CIY4 have QD symmetry, the QD latitudes of $\pm\, 55^{\circ }$ and $0^\circ$ are shown in red and blue, respectively. As expected, the top four maps show opposing streamlines mostly following QD lines of latitude and the two major solar-quiet (Sq) foci remaining mostly aligned along the same meridian during vernal equinox.

The bottom four maps show Sq foci aligned along the same meridian during vernal (March) and autumnal (September) equinox, while the northern foci is stronger and lags the southern foci in local time during northern summer (June) and the opposite happening during northern winter (December). Hence, the maps are similar to those from Sabaka et al. (2002, 2015); Chulliat et al. (2016) and are realistic at low and mid-latitudes. At high latitudes, however, the field is probably over damped and does not show the fixed-local time cells related to the well-known current systems associated with plasma convection in the polar cap ionosphere, for example captured in the SIFMplus model of Olsen et al. (2016).

In order to further validate the CIY4 ionospheric field, a comparison of predictions of QD mid-latitude OHM values was performed between CIY4 and a hybrid model in which the CIY4 ionosphere was replaced by the *Swarm* L2 “Dedicated Ionospheric Field Inversion” (DIFI) product presented in Chulliat et al. (2016), but updated with data through 2017 (*Swarm* L2 product SW_OPER_MIO_SHA_2D_20131201T000000_20171231T235959_0402), and the magnetosphere was replaced by the *Swarm* L2 MMA product (SW_OPER_MMA _SHA_2C_20131201T000000_20180101T000000_0401, described in the next section). The weighted RMS fit, $r_w$, in the *NEC* frame from the hybrid model is (5.903, 6.056, 5.024) nT for dayside data and (3.512, 3.809, 3.657) nT for nightside data. This can be compared to the values for CIY4 from Table 6 in which the dayside is (6.861, 7.882, 5.452) nT and nightside is (4.133, 4.847, 3.675) nT. The hybrid model is clearly out performing CIY4 for this data set on the basis of $r_w$, which is due to the DIFI ionospheric field predicting these data more closely. However, it should be stressed that the goal of field modeling is not the fitting of data, but rather the extraction of the most plausible geophysical parameters.

**Fig. 13:**
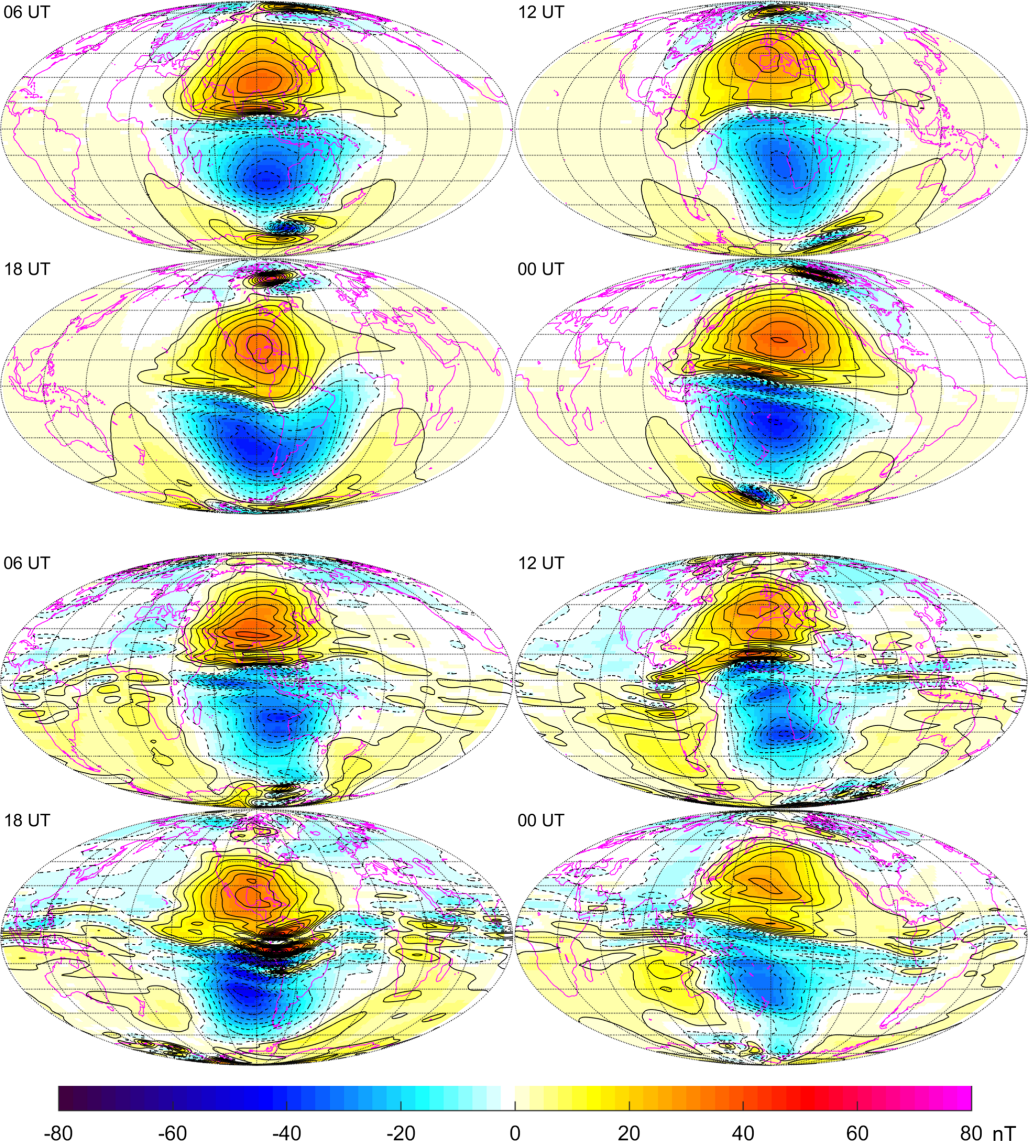
The top four maps show the radial component of the primary ionospheric magnetic field from the CIY4 model at Earth’s surface during vernal equinox centered on noon local time for 06, 12, 18, and $00\,\hbox {UT}$. The bottom four maps are similar to the top, but from the *Swarm* L2 Dedicated Ionospheric Field Inversion (DIFI) product (see Chulliat et al. 2016). Solid/dashed contour lines indicate positive/negative contours in increments of $5\,\hbox {nT}$. A value of $F_{10.7}=100.0\times 10^{-22}\,\mathrm{W}/\mathrm{m}^2/\mathrm{Hz}$ was used for evaluation

To illustrate this point, Fig. 13 shows maps of the radial component of the primary ionospheric magnetic field from CIY4 and from DIFI at Earth’s surface during vernal equinox centered on noon local time for 06, 12, 18, and $00\,\hbox {UT}$. It is apparent that the large-scale Sq vortices are in generally good agreement with respect to position and strength. However, the DIFI fields exhibit much more small-scale structure, which is undoubtably allowing for better data fits, at least for the QD mid-latitude OHM data. It certainly could be that the CIY4 ionospheric field is overly smooth, as alluded to in section “Parameter separability,” but it could also be that some of the small-scale structure in DIFI is spurious. Indeed, the true state may lie between the two extremes.

### Magnetospheric and induced fields

The CIY4 model is based on magnetic field observations from geomagnetic quiet periods, and as described in section “Magnetospheric field,” degree-1 external (magnetospheric) and internal (induced) SH expansions are co-estimated in hourly bins for the selected quiet periods. However, in order to obtain a continuous time series of magnetospheric and induced expansion coefficients a subsequent non-comprehensive approach is used: first, remove the CIY4 models of core, lithosphere (including observatory biases when applicable) and ionosphere (and its secondary induced part) from magnetic observations taken by *Swarm* and ground observatories covering the whole period from December 2013 to December 2017, including the geomagnetic disturbed periods that were excluded from CIY4, and then perform a SH analysis of the residuals in bins of 1.5 and 6 h duration for degree-1 and higher degree coefficients, respectively. Details of this resulting *Swarm* MMA (“Magnetic-Magnetospheric”) L2 product will be described in a separate publication.

**Fig. 14:**
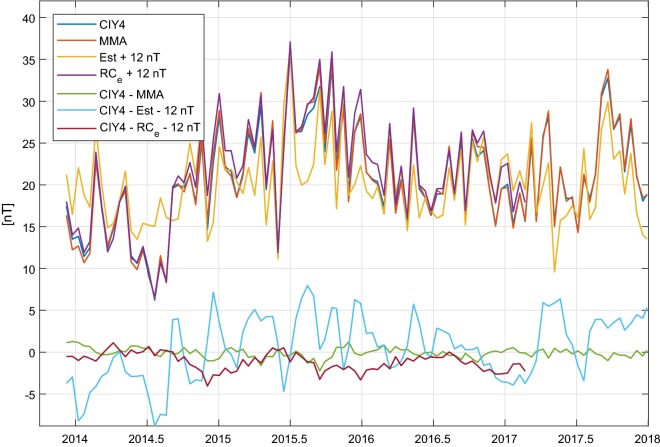
The top set of curves shows the 15-day averages of $q_1^0$ as estimated in CIY4 (blue curve), in comparison with the corresponding coefficient from the *Swarm* MMA data product (red), $-Est$ (yellow), and $-RC_e$ (purple). The bottom set of curves shows the differences of the various estimates. See text for details

The CIY4 estimates of the dominant magnetospheric coefficient $q_1^0$ are now assessed by comparing 15-day averages of the CIY4 estimates with 15-day averages of other values, including MMA, selected for the quiet periods for which CIY4 values are available. The top set of curves in Fig. 14 shows the excellent agreement between $q_1^0$ as determined by CIY4 (blue curve) and MMA (red); the difference between the two values (green) is less than $1{-}2\,\hbox {nT}$. Also shown is $RC_e$ (purple curve), which is the external, magnetospheric part of *RC*, an index of magnetospheric ring-current strength (Olsen et al. 2014) determined using 14 ground magnetic observatories (in the reference, 21 observatories were used to define *RC*), and *Est*, which is the external part of the *Dst* index determined using data from four low-latitude magnetic observatories (Maus and Weidelt 2004). Agreement between CIY4 and $-RC_e$ (the negative sign makes the value comparable with $q_1^0$) is also very good; their difference (dark red curve) is smaller than $3\,\hbox {nT}$ after correction for an offset in $-RC_e$ of $12\,\hbox {nT}$. This offset accounts for the unknown absolute baseline level of ring-current indices such as *RC* and *Dst*, which are entirely determined from ground observatory data. There seems to be a small annual variation in the difference of about $1\,\hbox {nT}$ amplitude, with minima in December and maxima in June, whose origin is unknown. The difference with $-Est$ (light blue curve) reveals erratic variations of up to $\pm \, 8\,\hbox {nT}$ and more, which reflects the well-known baseline-instabilities of the *Dst* index (e.g., Olsen et al. 2014).

## Conclusions

The ESA *Swarm* L2 CI magnetic products have been extracted from the CIY4 parent model that was produced from 4 years of *Swarm* satellite and complementary observatory hourly means data. The core, lithospheric, ionospheric, and magnetospheric fields, as well as the new $M_2$ tidal product, have been validated and are found to be of good quality. The core field is in good agreement with the CHAOS-6 model, and the modeled SV follows closely trends seen at ground observatories. The SV in CIY4 is stable at the CMB out to at least degree 13, with a region of rapid change in core field SA seen at low latitudes under the Eastern Pacific and South America between 2014 and 2018. The lithospheric field agrees quite well with the MF7 model and the new high-resolution LCS-1 model over the entire SH degree range $n=15{-}100$. Maps of the radial field show good agreement, even at high latitudes. The power in the differences between CIY4 and these models is still well below the power of the actual lithospheric field over this same SH degree range. The ionospheric field at low-to-mid-latitudes is also plausible and exhibits the same large-scale structure as seen in previous CMs and the DI versions. The LT variability of its stream function as a function of UT and season is also what is expected. The estimated quiet-time magnetospheric field variation shows good agreement with independent estimates of magnetospheric ring-current activity like *RC* and *Dst*.

The new *Swarm* $M_2$ magnetic field product has been introduced in this paper. Its field coefficients will be distributed in real rather than complex form, and thus, there will be 2 coefficients for $m=0$ terms and 4 for $m{>}0$ terms. The progression from CHAMP through 2, 3, and now 4 years of *Swarm* data, culminating in the CIY4 model, shows a clear evolution of improvement in resolving the oceanic $M_2$ magnetic field signal. Given that the CHAMP and *Swarm* missions are independent and have flown at different times under different conditions, the agreement between their $M_2$ fields in amplitude and phase is very impressive. The resolution achieved with *Swarm* also suggests that other major tidal constituents could be convincingly detected.

## Abbreviations

CI: comprehensive inversion
CM: comprehensive model
CMB: core–mantle boundary
CRF: common reference frame
DI: dedicated inversion
DIFI: Dedicated Ionospheric Field Inversion
DISC: Data Innovation and Science Center
ESA: European Space Agency
EW: east–west
ECEF: Earth-Centered Earth Fixed
GN: Gauss–Newton
LS: least squares
LSLE-GN: least squares linear equality Gauss–Newton
L1b: level-1b
L2: level-2
LT: local time
MMA: magnetic-magnetospheric
NS: north–south
NEC: north, east, center
OHM: observatory hourly means
QD: quasi-dipole
SCARF: Satellite Constellation Application and Research Facility
SIVW: Selective Infinite Variance Weighting
SA: secular acceleration
SH: spherical harmonic
SV: secular variation
Sq: solar-quiet
UT: universal time
VMF: vector magnetometer frame
1D: one-dimensional
3D: three-dimensional
